# YBX1 regulates RNA polymerase III transcripts to prevent inflammation

**DOI:** 10.1073/pnas.2605741123

**Published:** 2026-08-21

**Authors:** Tania I. Strilets, Sarah E. Dremel, Mariano A. Garcia-Blanco

**Affiliations:** ^a^Department of Biochemistry and Molecular Biology, The University of Texas Medical Branch, Galveston, TX 77555; ^b^ Department of Microbiology, Immunology and Cancer Biology, University of Virginia, Charlottesville, VA 22908; ^c^ Center for RNA Science and Medicine, University of Virginia, Charlottesville, VA 22908

**Keywords:** RNA, YBX1, immunity, inflammation, RNA polymerase III

## Abstract

Tolerance of endogenous nucleic acids is crucial for cellular homeostasis and when perturbed can lead to immune dysregulation. RNA-binding proteins (RBPs) bind, modify, and compartmentalize endogenous RNAs to prevent their recognition by the innate immune system. We found that loss of a highly abundant RBP, the cold shock domain containing Y-box binding protein 1 (YBX1), results in the spontaneous induction of an IL6/STAT3 inflammatory response and the upregulation of interferon-stimulated genes. A meta-analysis of other studies reveals that the depletion of YBX1 orthologs and paralogs results in a similar proinflammatory gene signature, suggesting a conserved role of Y-box proteins in maintaining innate immune homeostasis. Among RNAs that interact with YBX1, we tested whether endogenous RNA polymerase III (RNAP III) transcripts stimulate innate immune signaling when YBX1 is depleted. We demonstrate that the inflammatory responses induced by the loss of YBX1 are abrogated by inhibiting RNAP III transcription and RIG-I signaling. Hence, we hypothesize that by regulating RNAP III transcripts, YBX1 prevents their recognition by the innate immune system, uncovering a role of this RBP in maintaining cellular homeostasis and tolerance to endogenous RNAs. The conservation of this phenotype across YBX1 orthologs and paralogs argues that RNA shielding by cold shock domain proteins represents an evolutionarily conserved solution to the problem of endogenous RNA immunogenicity.

## Significance

Endogenous nucleic acids, including DNA and RNA, possess immunostimulatory features that, under normal cellular conditions, remain contained. Cellular stress, injury, or pathogenic infection releases endogenous nucleic acids to trigger innate immune responses, transforming them into damage-associated molecular patterns (DAMPs). Thus, cells allocate significant resources to shielding endogenous nucleic acids from the innate immune system. One such example includes the sequestration of noncoding RNAs by RNA-binding proteins (RBPs). Here, we report that a ubiquitous RBP, Y-box binding protein 1 (YBX1), prevents noncoding RNAs synthesized by RNA polymerase III from being recognized as DAMPs under cellular homeostasis. Together, our results uncover an underappreciated role for YBX1 as a guardian of noncoding RNA homeostasis, preventing inappropriate activation of innate immunity.

Cellular homeostasis involves the proper synthesis, processing, turnover, and localization of endogenous macromolecules, including nucleic acids, proteins, and fatty acids. When these processes are dysregulated by cellular injury, stress, or infection, endogenous molecules can be recognized by the innate immune system, transforming them into damage-associated molecular patterns (DAMPs) (1). For example, mislocalization of mitochondrial DNA into the cytoplasm triggers the potent activation of cyclic GMP-AMP synthase (cGAS)–dependent inflammatory responses (2). Endogenous RNAs can also trigger inflammatory responses and contribute to disease if not properly localized or processed (1, 3–5); for instance, editing of adenosines to inosines by ADAR1 or removal of 5′ triphosphates by DUSP11 (6, 7). Many endogenous RNAs that serve as DAMPs are RNA polymerase III (RNAP III) transcripts, including Alu elements, 7SL, Y, vault, and 5S ribosomal pseudogene RNAs (8–17). For example, Alu RNAs can stimulate the innate immune system by binding and activating RIG-I-like receptors (RLRs), endosomal Toll-like receptors (TLRs), and the NLRC4 inflammasome (7, 8, 16, 18, 19). Interaction of RNA-binding proteins (RBPs) with endogenous noncoding RNAs has revealed itself as a regulatory mechanism to prevent their recognition as DAMPs. Examples of RBPs that do so to prevent inflammatory responses include Ro60, TDP-43, and HNRNPC (8, 18, 19). Notably, YBX1’s interactions with RNAP III transcripts are well documented; YBX1 binds tRNAs, tRNA-derived fragments, 7SK, and Y RNAs (20–22). Moreover, YBX1 sorts RNAP III transcripts like tRNAs, vault, and Y RNAs into extracellular vesicles (23). However, the biological consequences of YBX1’s interactions with these endogenous noncoding RNAs remain largely unexplored.

Here, we report that the highly conserved cold shock domain (CSD) containing RBP, Y-box binding protein 1 (YBX1), is a crucial effector of cellular homeostasis. Depletion of YBX1 leads to a strong proinflammatory signature in cells, characterized by the transcriptional upregulation and secretion of IL6 and STAT3 phosphorylation. Specifically, we identified that BRF1-dependent RNAP III transcripts contributed to the induction of inflammation observed with YBX1 depletion, suggesting that these endogenous noncoding RNAs serve as DAMPs in this context. As observed with other endogenous immunostimulatory RNAP III transcripts, the induction of inflammation associated with YBX1 depended on RLR signaling (4). Thus, we posit that YBX1 has a fundamental function in sustaining cellular noncoding RNA homeostasis by preventing the innate immune system from recognizing RNAP III transcripts. Our meta-analysis of other studies reveals similar proinflammatory signatures in mouse cells depleted of YBX1 and human cells depleted of Y-box binding protein 3 (YBX3). Together, these findings implicate a conserved function of Y-box binding proteins in preventing aberrant activation of the innate immune system.

## Results

### Knockout of YBX1 Induces IL6 Secretion and Activates STAT3 Signaling.

**Fig. 1.**
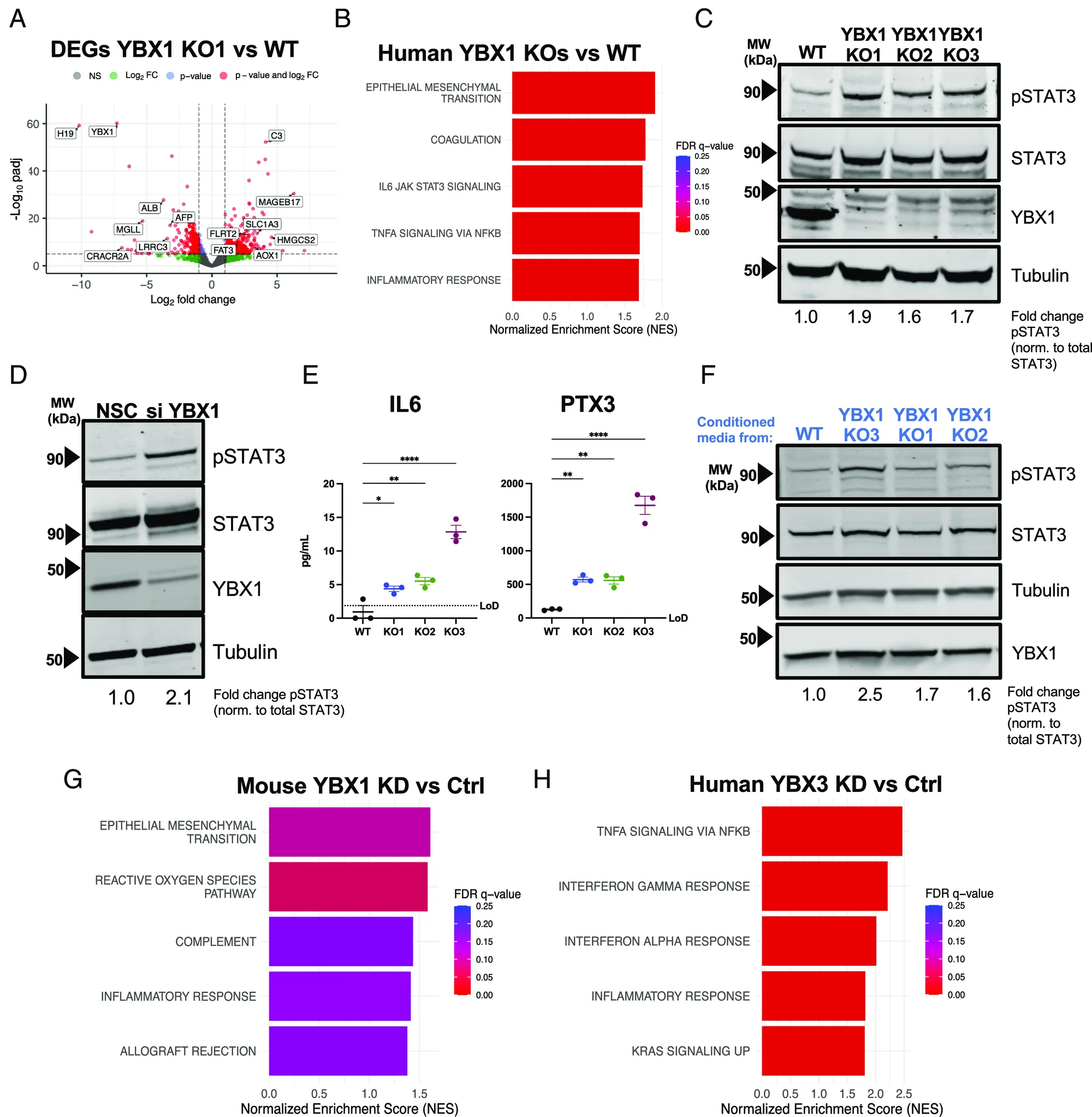
Depletion of YBXs leads to upregulation of proinflammatory transcripts across species. (*A*) Volcano plot of DEGs in Huh7 YBX1 knock-out clone 1 (KO1) vs. WT cells, analyzed using DeSeq2. The top seven upregulated and downregulated genes common to two YBX1 KO clones are annotated. Log2 fold change (Log2FC) cutoff = 1, adjusted *P*-value cutoff = ≤1 × 10^−5^. (*B*) Top five MSigDB Hallmark pathway gene sets enriched in YBX1 KO cells relative to WT cells, analyzed via GSEA. All gene sets in this figure were ranked by normalized enrichment score (NES) and visualized using the FDR q-value. (*C*) Western blot of phosphorylated STAT3 (pSTAT3), total STAT3, YBX1, and Tubulin protein levels in Huh7 WT and three YBX1 KO clonal cell lines (KO1, KO2, and KO3). Band intensity of pSTAT3 was determined by densitometric analysis in ImageJ, normalized to total STAT3, and expressed as fold change relative to WT cells. Data are from one representative experiment. (*D*) Same as *C* but in Huh7 WT cells treated with nonsilencing control (NSC) siRNA or an siRNA against YBX1 (si YBX1). Band intensities of pSTAT3 are displayed as fold-change levels relative to NSC cells. Data are from one representative experiment. (*E*) Multiplexed immunoassay results of conditioned media harvested from Huh7 WT and YBX1 KO cells to detect secreted cytokines and proinflammatory mediators. *P < 0.05, **P < 0.005, ***P < 0.0005, ****P < 0.00005, ns= not significant. Lines represent mean ± SEM. LoD = Limit of detection. pg/mL = picograms per mL. Data represent triplicate values from one independent experiment. (*F*) Conditioned media from Huh7 WT and YBX1 KO cells was used to culture Huh7 WT cells for 4 h before harvesting cells for western blot to assay STAT3 phosphorylation. Band intensity of pSTAT3, shown as fold change relative to WT cells. Data are from one representative experiment. (*G*) Top five MSigDB Hallmark pathway gene sets enriched in murine BaF3 V617F cells treated with shRNAs against YBX1 relative to shNT control cells from Jayavelu et al. (28). Log2FC values of DEGs in YBX1 shRNA-treated cells relative to shNT were analyzed via preranked GSEA. (*H*) Same as *G* but for HeLa cells treated with siRNAs against YBX3 relative to KD control cells from Cooke et al. (29).

Given the well-documented role for YBX1 in RNA transactions, we examined the effect of YBX1 KO on global gene expression using Illumina-based RNA-Sequencing (24). First, using DESeq2, we compared differentially expressed genes (DEGs) between the parental wild type (WT) Huh7 cells used to generate YBX1 KO clones (WT, n = 2) and two independently generated YBX1 KO clonal cell lines (YBX1 KO1; n = 3, and YBX1 KO3; n = 3) (25). YBX1 KO1 led to many DEGs in both clones (Fig. 1*A* and KO3 in *SI Appendix*, Fig. S1*A*). Next, to better understand the broad cellular processes altered in YBX1 KO3 and KO1 cells, we performed gene set enrichment analysis (GSEA) using the molecular signatures database (MSigDB) hallmark gene sets (26, 27). Gene sets with a false discovery rate (FDR) q-value cutoff of ≤0.25 were considered significantly enriched, and those with an FDR q-value of ≤0.05 were considered highly significantly enriched. Intriguingly, the five top gene sets enriched in YBX1 KO3 and KO1 are related to proinflammatory processes (Fig. 1*B* and Dataset S1). For instance, YBX1 KO3 and KO1 cells display the enrichment of hallmark gene sets involved in the IL6/STAT3 and TNFα/NF-κB signaling pathways (*SI Appendix*, Figs. S2–S4 and Dataset S1). Transcripts whose expression was altered in YBX1 KO1 and KO3 and contributed to the core enrichment of these gene sets included *TLR2*, *LIF*, *PTX3*, and *IL6* (*SI Appendix*, Figs. S2 and S3 and Dataset S1). Heatmaps displaying gene expression patterns of all transcripts contributing to the positive enrichment of the hallmark inflammatory gene sets are shown in *SI Appendix*, Figs. S2–S4.

The top canonical inflammatory gene set enriched in both YBX1 KOs is the Hallmark IL6 JAK STAT3 signaling gene set (Fig. 1*B*) (27). To confirm these findings biochemically, we assessed the phosphorylation status of STAT3 (pSTAT3) across three YBX1 KO clonal cell lines (KO1, KO2, KO2), relative to the parental WT cells and observed an average 1.7-fold (±0.2 SD) increase in pSTAT3 (Fig. 1*C*). This phenotype was also seen in transient depletion of YBX1 in the parental WT cells using two independent siRNAs (Fig. 1*D* and *SI Appendix*, Fig. S1*B*) Thus, in the absence of exogenous stimuli loss of YBX1 induced an inflammatory state in cells.

Since YBX1 KO cells had gene expression and signaling signatures of inflammation, we asked whether these cells were secreting proinflammatory cytokines/mediators. Using multiplexed immunoassay analysis, we observed that YBX1 KO cells (KO1, KO2, KO3) secrete significantly higher amounts of IL6 and PTX3 into cell culture media relative to parental WT cells (Fig. 1*E*). The secretion of other proinflammatory cytokines and mediators identified in the GSEA, like C3, IL8, and IL-1β, was not stimulated by YBX1 KO revealing differences between the transcriptomic upregulation of proinflammatory mediators and their secretion (*SI Appendix*, Fig. S5*A*). Despite the enrichment of the TNFA SIGNALING VIA NFKB gene set, we could not detect the secretion of TNFα by YBX1 KO cells by multiplexed immunoassay (*SI Appendix*, Fig. S6). To test if the cytokines secreted by YBX1 KO cells could induce STAT3 signaling in a paracrine manner, we cultured parental WT Huh7 cells with conditioned media from WT and YBX1 KO cells for 4 h, and assayed pSTAT3 protein levels. Cells cultured with media from the three YBX1 KO clones (KO1, KO2, KO3) had an average of 1.9-fold (±0.5 SD) higher level of pSTAT3 than those cultured with conditioned media from WT cells (Fig. 1*F*). This increase in pSTAT3 is observed for up to 24 h of conditioned media treatment (*SI Appendix*, Fig. S5*B*). This confirmed that loss of YBX1 induced a dysregulation of cellular homeostasis, characterized by secretion of proinflammatory cytokines like IL6, and activation of STAT3 signaling.

### Across Orthologs and Paralogs, Depletion of Y-box Binding Proteins Is Associated with Inflammation.

We turned to the literature to investigate if this pro-inflammatory phenotype was specific to our YBX1 KO cells. YBX1 has orthologs across various species and two human paralogs (YBX2 and YBX3) with high amino acid similarity in the cold-shock domain (30). Previously, Jayavelu et al. performed RNA-Seq of mouse cells depleted of YBX1 by short-hairpin RNAs (shRNAs) (28). They demonstrate the enrichment of Gene Ontology (GO) terms associated with inflammation during YBX1 knockdown (KD), which is 96% similar in amino acid composition to human YBX1 (30). To explore overlap in the inflammatory phenotypes with our data, we performed a cross-species GSEA of DEGs identified during YBX1 KD in mouse cells. Our meta-analysis identified the enrichment of hallmark gene sets involved in inflammation (Fig. 1*G* and Dataset S2). Transcripts enriched during murine YBX1 KD contributing to the core enrichment of the hallmark “inflammatory response” gene set included those encoding cytokines like IL1β, IL1α, and IL6 (Dataset S2). Similarly, we performed GSEA analysis of DEGs identified in YBX3-depleted HeLa cells from Cooke et al. for a cross-paralog comparison (29). This analysis also displays the enrichment of gene sets associated with inflammation in YBX3-depleted cells, including those related to TNFα/NF-κB signaling, similar to our YBX1 KO results (Fig. 1*H* and Dataset S3). However, unlike our YBX1 KO cells, YBX3 KD cells also display the enrichment of gene sets associated with a type I interferon (IFN) response (Fig. 1*H*). Together, these analyses display that across orthologs and paralogs, Y-box binding proteins prevent the induction of a proinflammatory state in cells. This suggests a conserved role of these RBPs in maintaining cellular homeostasis.

### YBX1 Suppresses Inappropriate Activation of Diverse Immune and Inflammatory Pathways.

**Fig. 2.**
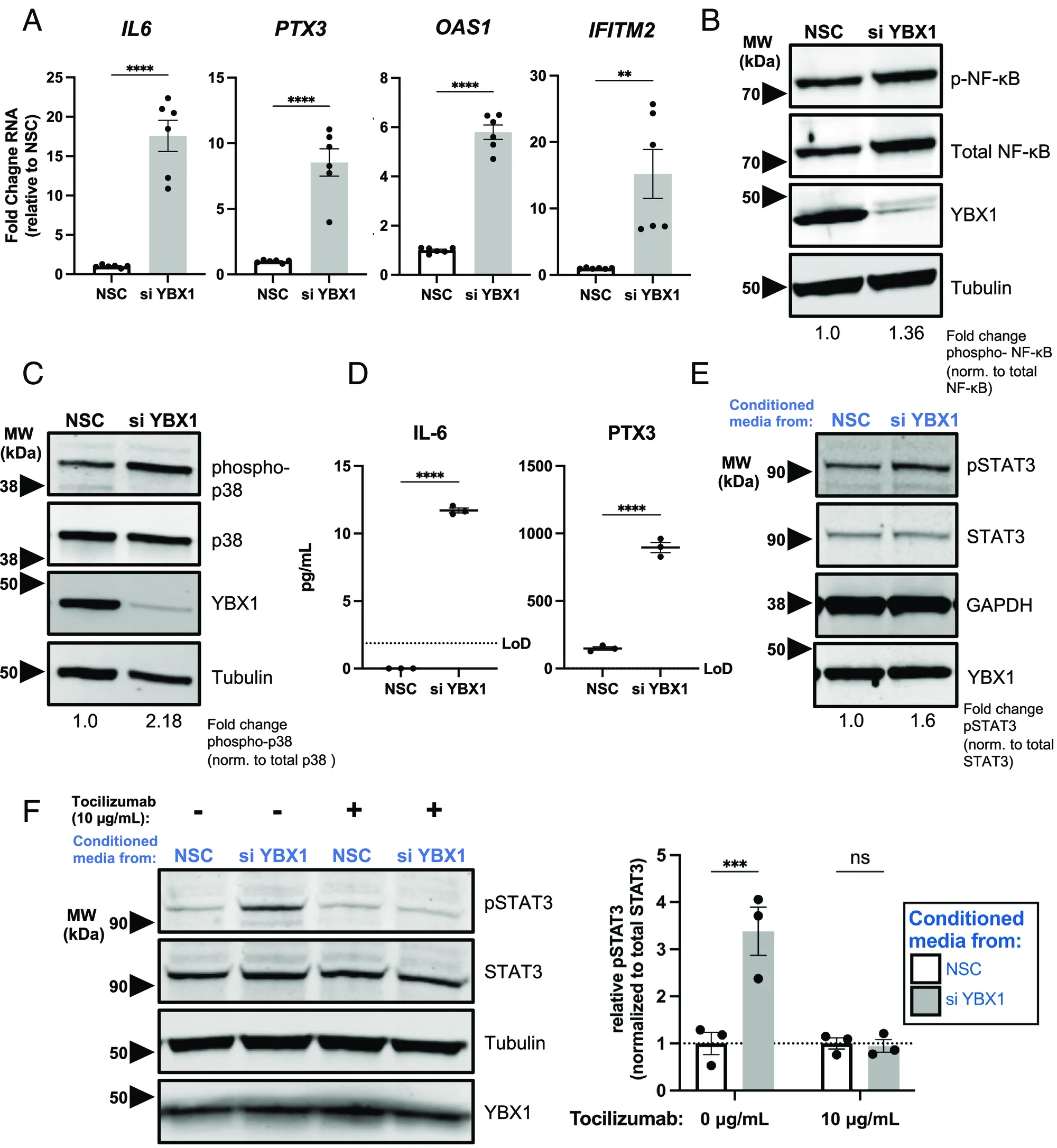
YBX1 prevents the activation of IL6/STAT3 immune response in the absence of exogenous stimuli. (*A*) qRT-PCR of select proinflammatory transcripts (*IL6, PTX3, OAS1, and IFITM2*) identified upregulated in YBX1 KO cells via RNA-Seq during YBX1 KD in Huh7 WT cells. Huh7 WT cells were treated with NSC siRNA or an siRNA against YBX1. Transcripts were normalized to *GAPDH* and expressed as fold change relative to WT cells. Data represent triplicate values from two independent experiments. Each point represents one experimental sample. For all bar and scatter plots in this figure, *P < 0.05, **P < 0.005, ***P < 0.0005, ****P < 0.00005, ns= not significant. Bars represent mean ± SEM. (*B*) Western blot of phosphorylated NF-κB (phospho- NF-κB), total NF-κB, tubulin, and YBX1 protein levels in Huh7 WT cells treated with NSC siRNA or an siRNA against YBX1 (si YBX1). NF-κB band intensity was determined by densitometric analysis in ImageJ, normalized to total NF-κB, and expressed as fold change relative to NSC-treated cells. Data are from one representative experiment. (*C*) Same as in *B* but for phosphorylated p38 (phos- p38) and total p38. Data are from one representative experiment. (*D*) Multiplexed immunoassay results of conditioned media harvested from Huh7 WT cells treated with NSC siRNA or an siRNA against YBX1 (si YBX1) to detect secreted cytokines and proinflammatory mediators. LoD = Limit of detection. pg/mL = picograms per mL. Data represent triplicate values from one independent experiment. (*E*) Conditioned media from Huh7 WT cells treated with NSC siRNA or an siRNA against YBX1 (si YBX1) were used to culture Huh7 WT cells for 4 h before harvesting cells for western blot to assay STAT3 phosphorylation. Band intensity of pSTAT3 was determined by densitometric analysis in ImageJ, normalized to total STAT3, and expressed as fold change relative to cells with NSC-conditioned media added. Data are from one representative experiment. (*F*) Huh7 cells were pretreated with an anti-IL6 receptor antibody (Tocilizumab, 10 µg/mL) for 1 h at 37 °C before the addition of conditioned media from Huh7 WT cells treated with a NSC siRNA or an siRNA against YBX1 (si YBX1), and processed for western blot of pSTAT3, total STAT3, Tubulin, and YBX1. Western blot data are from one representative experiment. Band intensity of pSTAT3 was determined by densitometric analysis in ImageJ, normalized to total STAT3, and expressed as fold change relative to cells with NSC-conditioned media added. Quantified pSTAT3 data are from three independent experiments.

Long-term depletion of YBX1 is likely to cause secondary adaptations that could mask a comprehensive view of the proinflammatory pathways activated in the absence of YBX1. To remedy this, we carried out short-term YBX1 depletion using siRNAs and interrogated inflammatory pathways. As shown above, YBX1 KD resulted in activation of STAT3 (Fig. 1*D* and *SI Appendix*, Fig. S1*B*). Additionally, with YBX1 KD we noted the upregulation of the same proinflammatory transcripts observed in YBX1 KO cells, like *IL6* and *PTX3* (Fig. 2*A* and *SI Appendix*, Fig. S7*A*). Unlike YBX1 KO; however, KD of YBX1 resulted in the transcriptional upregulation of IFN-stimulated genes like *OAS1* and *IFITM2* (Fig. 2*A* and *SI Appendix*, Fig. S7*A*). Moreover, we observed a 1.4-fold increase in phosphorylated NF-κB p65, and 2.2-fold increase in phosphorylated p38 a mitogen-activated protein kinase (MAPK) activated by immune stimulation and stress (Fig. 2 *B* and *C* and *SI Appendix*, Fig. S7*B*), (31). However, YBX1 KD did not induce the phosphorylation of STAT1, suggesting that the dominant inflammatory response in these cells is likely not mediated through Type I IFN induction (*SI Appendix*, Fig. S7*C*). Further, these results are not cell line specific, as YBX1 KD in A549 cells also resulted in a similar broad proinflammatory phenotype (*SI Appendix*, Fig. S7 *D* and *E*).

We confirmed that, like YBX1 KOs, YBX1 KD cells secreted comparable levels of IL6 and PTX3 into cell culture media (Fig. 2*C*). 4-h treatment of conditioned media from YBX1 KD cells induced 1.6-fold more pSTAT3 in WT Huh7 cells relative to conditioned media from the NSC siRNA treated cells (Fig. 2*D*). As pSTAT3 can also be induced through other cytokines, including TNF family members and Type I IFN, we assessed the contribution of secreted IL6 toward pSTAT3 induction by IL6 receptor blockade using the humanized monoclonal antibody, Tocilizumab. Preincubation of Huh7 WT cells with Tocilizumab prior to a 4-h challenge with recombinant human IL6 protein reduced pSTAT3 induction by ~1.6 fold (±0.28 SD), quantified via western blot (*SI Appendix*, Fig. S8), indicating Tocilizumab efficacy in our experimental system. Importantly, when conditioned media from YBX1 KD cells was applied to Tocilizumab-treated Huh7 WT cells, pSTAT3 induction was abrogated to levels similar to NSC conditioned media-treated cells (Fig. 2*F*). These data demonstrate that in YBX1 KD conditions, IL6 is the major contributor to the proinflammatory pSTAT3 phenotype observed.

YBX1 KO cells also displayed increased expression of AXL protein, a receptor tyrosine kinase involved in the negative regulation of type I IFN-mediated innate immune responses (*SI Appendix*, Fig. S9*A*) (32). We also confirmed that there is no activation of a type I IFN or double-stranded RNA response in YBX1 KO cells, as evidenced by no differences in the transcriptional levels of *OAS1* between WT cells and YBX1 KO clones (*SI Appendix*, Fig. S9*B*). YBX1 KO clones also did not display the activation of STAT1 or PKR phosphorylation (*SI Appendix*, Fig. S9 *C* and *D*). Furthermore, YBX1 KOs had a ~twofold increase in YBX3 protein levels (*SI Appendix*, Fig. S9*E*), like others have demonstrated in YBX1 KO HEK 293 T cells (33).

Overall, we observe pleiotropic responses to YBX1 depletion by KD and KO; however, both are dominated by the activation of an IL6/pSTAT3 phenotype. Short-term YBX1 depletion with siRNAs leads to phosphorylation/activation of NF-κB and p38, resulting in transcription of proinflammatory cytokines and secretion of IL-6/PTX3. Furthermore, STAT3 phosphorylation/activation depends on IL6 secretion. Since this pleiotropic response occurs in the absence of external stimuli, we posit that YBX1 plays a crucial role in cellular homeostasis by preventing the innate immune system from sensing DAMPs.

### RNAP III Transcripts Serve as DAMPs During YBX1 Depletion.

**Fig. 3.**
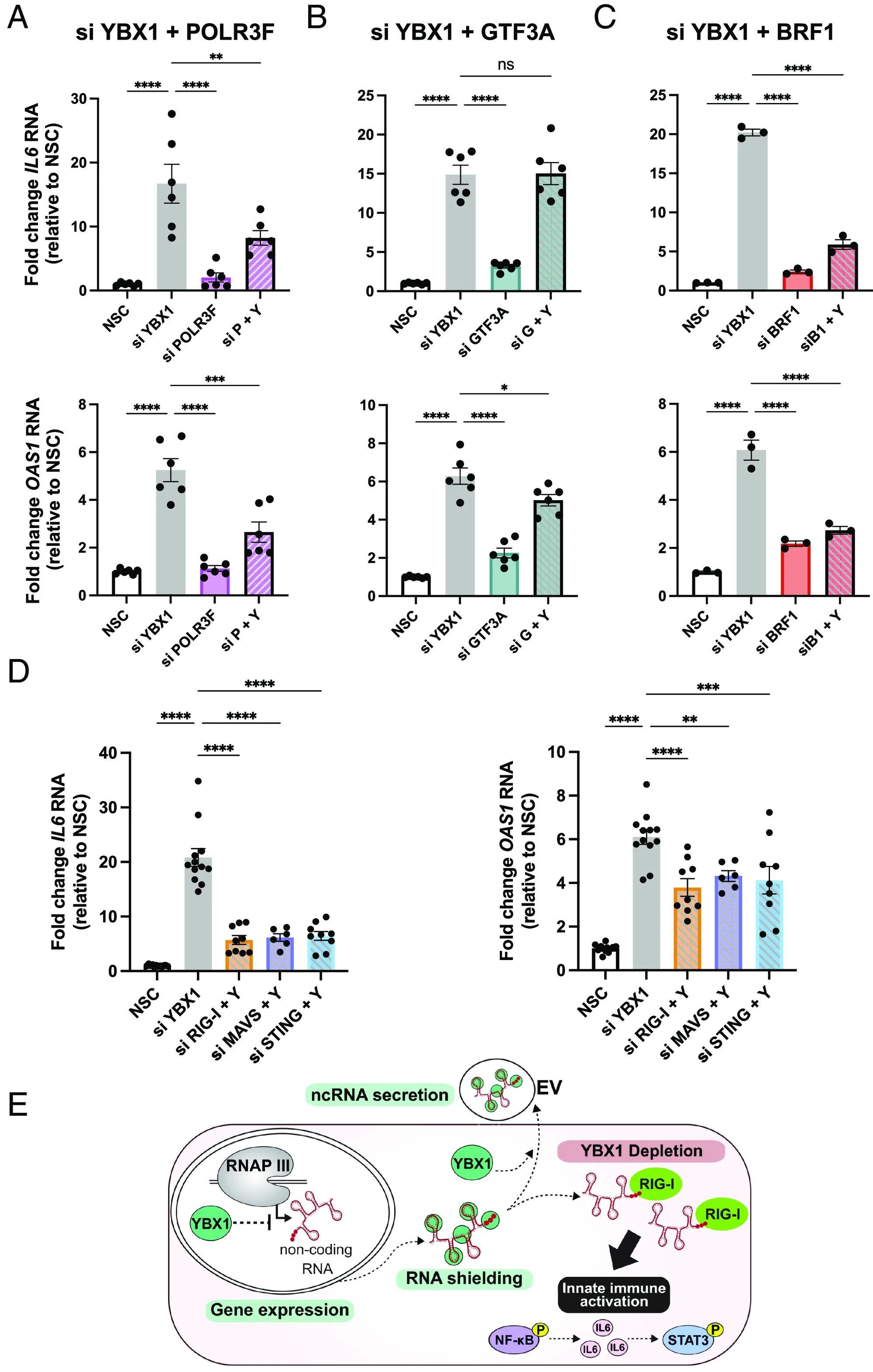
YBX1 prevents the activation of RLR signaling by RNAP III transcripts. (*A–C*, *upper row*) qRT-PCR assaying the upregulation of *IL6* during simultaneous KD of YBX1 and RNAP III machinery. Huh7 WT cells were treated with NSC siRNA, an siRNA against YBX1 (si YBX1), siRNA pools against POLR3F (magenta), GTF3A (teal), or BRF1 (red), or a combination of si YBX1 and siRNA pools against POLR3F, GTF3A, and BRF1. For all qRT-PCR data in this figure, transcript abundance was normalized to *GAPDH* and expressed as fold change relative to NSC-treated cells. *P < 0.05, **P < 0.005, ***P < 0.0005, ****P < 0.00005, ns = not significant. Each point represents one experimental sample. Data are from one or two independent experiments. Bars represent mean ± SEM. (*A–C, bottom row*) Same as in *A*, but assaying *OAS1* transcripts. (*D*) qRT-PCR assaying the upregulation of *IL6* and OAS1 during simultaneous KD of YBX1 and innate immune signaling components. Huh7 WT cells were treated with NSC siRNA, an siRNA against YBX1 (si YBX1), or a combination of an siRNA against YBX1 and siRNA pools against RIG-I (orange), MAVS (purple), and STING (blue). Data are from two or three independent experiments. (*E*) Summary hypothesis of how YBX1 regulates RNAP III transcripts to maintain cellular homeostasis and prevent inflammation (*Discussion*). EVs = extracellular vesicles.

Next, we investigated the identity of the endogenous molecules that act as DAMPs upon YBX1 depletion to trigger inflammation. Our primary candidates were RNAs synthesized by RNAP III since these possess immunogenic moieties like double-stranded RNA regions and 5′ triphosphate groups, and serve as DAMPs when dysregulated (4, 34, 35). Moreover, YBX1 binds and sorts RNAP III transcripts into extracellular vesicles, and YBX1 KO cells display an intracellular increase of RNAP III transcripts (20–23). Thus, we hypothesized that RNAP III transcripts may serve as DAMPs “unshielded” to the innate immune system when YBX1 is depleted. To test this hypothesis, we performed simultaneous KD of both YBX1 and a core RNAP III subunit, POLR3F, whose KD has previously been shown to inhibit RNAP III transcription (12, 36). We confirmed successful inhibition of RNAP III transcription during POLR3F KD by assaying pre-tRNA-Tyr-GTA and *BCYRN1* transcript levels and monitoring POLR3F protein levels (*SI Appendix*, Fig. S10 *A* and *B*). Notably, KD of YBX1 and POLR3F together significantly reduced the transcriptional upregulation of *IL6* and *OAS1* induced by YBX1 KD alone (Fig. 3*A*; compare second and fourth bars, *SI Appendix*, Fig. S11). Thus, the transcriptional activity of RNAP III contributed to inflammation induced by YBX1 depletion, implicating these transcripts as potential DAMPs.

RNAP III genes are organized into three types, each defined by their promoter and transcription factor requirements (37). To better understand what type of RNAP III transcripts serve as DAMPs, we performed simultaneous KD of YBX1 and RNAP III type-specific transcription factors. Type 1 RNAP III transcripts are 5S rRNAs and uniquely require the transcription factor TFIIIA/GTF3A for their synthesis (37). 5S rRNA pseudogenes, transcribed by GTF3A, are immunogenic and upregulated upon Herpes Simplex Virus-1 infection and alcohol-associated hepatitis (38, 39). Thus, we performed simultaneous KD of both YBX1 and GTF3A. KD of GTF3A was validated by measuring *GTF3A* transcript abundance via RT-qPCR and assaying protein levels (*SI Appendix*, Fig. S10 *C* and *D*). We tested type 1-specific RNAP III transcription inhibition, as there was no significant decrease of a type 2 RNAP III transcript, pre-tRNA-Tyr-GTA (*SI Appendix*, Fig. S10*C*). Codepletion of YBX1 and GTF3A did not alter *IL6* upregulation, albeit we observed a slight reduction in *OAS1* upregulation (Fig. 3*B*; compare second and fourth bars. Therefore, in our experimental system, depletion of GTF3A did not completely rescue the inflammatory responses associated with YBX1 loss.

Examples of type 2 RNAP III transcripts include tRNAs, Alu elements, and vault RNAs, some of which have immunogenic properties (4, 37). Type 2 transcripts do not have any unique RNAP III transcription factors required for their synthesis (37). However, type 1 and 2, but not type 3 transcripts, require the transcription factor BRF1 for their synthesis (37). Thus, we performed simultaneous KD of YBX1 and BRF1. We confirmed KD of BRF1 by measuring *BRF1* abundance and a BRF1-dependent transcript (pre-tRNA-Tyr-GTA) via RT-qPCR (*SI Appendix*, Fig. S10*E*). We also confirmed type 2-specific RNAP III transcriptional inhibition, as during BRF1 KD, we observed a decrease in pre-tRNA-Tyr-GTA transcripts, but not *RMRP* (*SI Appendix*, Fig. S10*E*). Notably, BRF1 and YBX1 codepletion significantly reduced the upregulation of IL6 and OAS1 observed with YBX1 loss (Fig. 3*C*), mirroring our results with POLR3F KD. Therefore, our data indicate that type 2 RNAP III transcripts are major contributors to the inflammatory response observed with YBX1 depletion and serve as DAMPs. Type 3 RNAP III transcription specifically requires BRF2, but because of our inability to effectively deplete BRF2, we have not elucidated whether type 3 RNAP III transcripts contribute to the YBX1-loss-induced inflammatory phenotype.

### Proinflammatory Phenotype Induced by YBX1 Loss Signals through RIG-I.

When dysregulated, RNAP III transcripts can activate the innate immune system by binding and activating RIG-I (4). To elucidate if the inflammatory responses induced by YBX1-loss were activated by a similar innate immune sensing mechanism, we performed simultaneous KD of YBX1 with RIG-I, MAVS, or STING (Fig. 3*D*). When bound to double-stranded RNA, RIG-I signals through the downstream adaptor mitochondrial antiviral signaling protein (MAVS) (40). Although STING is best known for sensing double-stranded DNA, (41–43), it also transduces innate immune signals during double-stranded RNA sensing by RIG-I (44). As expected, KD of YBX1 with all three of these innate immune signaling components robustly reduced the upregulation of *IL6* associated with YBX1 KD, and to a lesser extent, *OAS-1* (Fig. 3*D* and *SI Appendix*, Fig. S12 for KD confirmation). In conclusion, we have identified a role of YBX1 in cellular homeostasis by regulating type 2 RNAP III transcripts from triggering aberrant RLR-dependent innate immune activation.

## Discussion

For endogenous RNAs not to be recognized by the innate immune system, their proper compartmentalization, processing, and binding by RBPs is crucial (4). We identified that RNAP III transcripts act as DAMPS when unmasked by YBX1 depletion, are sensed through a RIG-I/MAVS/STING innate immune signaling cascade and result in activation of pleiotropic innate immune and inflammatory pathways (Fig. 3). Together, our findings implicate YBX1 as a central regulator of endogenous noncoding RNA immunological tolerance, revealing an unappreciated layer of RNA-based innate immune control for this ubiquitous and highly conserved RBP.

Transcriptomic analysis revealed that loss of YBX1 is characterized by a proinflammatory signature, enriched in hallmark gene sets related to IL6/STAT3 and TNFα/NF-κB signaling pathways (Fig. 1*B*). These results align with other publications in which depletion of YBX1 orthologs and paralogs leads to proinflammatory transcriptomic signatures, suggesting an anti-inflammatory function of these proteins (Fig. 1 *C* and *D*) (28, 29, 45, 46). Loss of YBX1 via KO and KD induced the persistent transcription and secretion of IL6, which we posit occurs via NF-κB transcription factor activation, and ultimately leads to STAT3 phosphorylation (Figs. 1 and 2). The reason YBX1 KO cells may tolerate this persistent proinflammatory state is likely due to compensatory repression of type I IFN responses, possibly mediated by upregulated AXL, and partial functional redundancy conferred by overexpressed YBX3 (*SI Appendix*, Fig. S9 *A* and *E*). Moreover, the YBX1 KO cells in this study were generated in a hepatocellular carcinoma cell line (Huh7s). YBX1 KOs may be able to tolerate persistent IL6, not Type I IFN cytokine synthesis and secretion because of the known role of IL6 signaling in promoting cell survival and proliferation pathways in malignant cells, compared to the predominantly cytotoxic effects of Type I IFN signaling (47–49).

Further characterization of the proinflammatory state conferred by YBX1 loss revealed that it was dependent on RNAP III activity and required RIG-I/MAVS/STING innate immune sensors (Fig. 3). Thus, we posit that YBX1 guards innate immune homeostasis by preventing RNAP III transcripts from acting as DAMPS. This may occur in two possible and not mutually exclusive ways: first, by YBX1 acting as a general negative regulator of RNAP III transcription in the nucleus, and second, by YBX1 preventing the cytoplasmic accumulation of naked RNAP III transcripts, impeding their recognition as DAMPs by RIG-I (Fig. 3*E*). YBX1 could act as a negative regulator of RNAP III transcription, as studies suggest it functions as a transcription factor (30, 50–54). Although we cannot rule this out, we favor the second general function of YBX1 by which it fine-tunes the abundance of RNAP III transcripts in the cytoplasm to prevent the accumulation of immunostimulatory RNAs that are unbound from RBPs and free to activate innate immune sensors, like RIG-I.

YBX1 knock-out HEK 293 T cells display around a ~1.5-fold increase in intracellular RNAP III transcripts such as tRNAs, vault RNAs, and Y RNAs, consistent with their impaired export into extracellular vesicles (23). Therefore, secretion into extracellular vesicles by YBX1 may expel RNAP III transcripts with immunostimulatory moieties away from innate immune RNA sensors that reside typically in endosomes and the cytoplasm. Thus, the RNAP III-dependent inflammatory responses we observe with YBX1 depletion could result from dysregulation of this process. This may be similar to YBX1’s proposed function in maintaining mitochondrial homeostasis, where it transports miR223 from mitochondria into cytoplasmic condensates for eventual secretion into extracellular vesicles (55, 56). It may also be related to YBX1’s ability to sort dengue virus nucleocapsids to sites of envelopment for the secretion of infectious virions (57).

It is also possible that YBX1 directly “shields” immunogenic moieties like double-stranded RNA regions and RNA 5′ triphosphate groups from RIG-I, as demonstrated for other RNAP III RNA–RBP interactions (12, 13). YBX1 binds RNAP III transcripts with immunostimulatory properties, including tRNAs and their derived fragments, 7SK RNA, and Y RNAs (4, 20–22). In our experimental model, KD of a core subunit of RNAP III, POLR3F, robustly inhibited inflammatory responses associated with YBX1 loss (Fig. 3*A*). Further investigation of what types of RNAP III transcripts contribute to the proinflammatory phenotypes revealed that the RNAP III transcription factor BRF1 and to a lesser extent GTF3A are involved in this axis (Fig. 3 *B* and *C*). Unlike BRF1 KD, GTF3A KD only partially ablated proinflammatory phenotypes associated with YBX1 loss. This could result from incomplete inhibition of GTF3A-mediated transcription of immunostimulatory transcripts in our experimental system (38, 39). Finally, many of the noncoding RNAs that YBX1 binds, including tRNAs, contain the RNA modification 5-methylcytosine(m^5^C) (20, 58, 59). YBX1 has an increased affinity for m^5^C-modified RNA, which could aid YBX1 binding to RNAP III transcripts for “shielding” from the innate immune system (60–62).

Altogether, given the conserved roles of CSD proteins (including YBX1) during stress responses, the anti-inflammatory actions of Y-box proteins may be a general function of cold shock proteins in normal cellular physiology (63, 64). We propose that YBX1 is a central gatekeeper of noncoding RNA physiology, coordinating the abundance, localization, and immunological inertness of RNAP III transcripts. The functional conservation of CSDs in stress resistance in plants and bacteria further suggests that this prohomeostatic role of YBX1 is conserved in many kingdoms of life (65).

## Materials and Methods

### Cell Lines and Viruses.

WT and YBX1 KO Huh7 cells (JCRB0403) were from ref. 57. A549 cells were obtained from the American Type Culture Collection (ATCC). Both Huh7 and A549 cells were maintained in Dulbecco’s Modified Eagle medium (DMEM, Genesee Scientific) supplemented with 10% heat-inactivated fetal bovine serum (FBS) (Genessee Scientific) and 100 U/mL penicillin and 100 mg/mL streptomycin (P/S, Gibco, Waltham, MA) at 37 °C with 5% CO_2_. Dengue virus 2 New Guinea C strain was obtained from World Reference Center for Emerging Viruses and Arboviruses (WRCREVA) at the University of Texas Medical Branch (UTMB). Cells were tested for *Mycoplasma* using the MycoAlert kit (Lonza) and used for no more than 10 passages.

### siRNA and Poly I:C Transfection.

siRNAs and final concentrations used in this study are detailed in *SI Appendix*, Fig. S13*A*. For all siRNA transfections, the nonsilencing negative control siRNA (Thermo Fisher Scientific) was used at the highest total nanomolar concentration as targeting siRNAs. siRNA complexes were reverse-transfected using 1 μL of Lipofectamine RNAiMAX (Thermo Fisher Scientific) per reaction, into 1 × 10^5^ Huh7 or A549 cells in a 24-well plate format, according to the manufacturer’s instructions. siRNA complexes were assembled in OptiMEM (Thermo Fisher Scientific). Cells used for siRNA transfections were maintained in DMEM (Genesee Scientific) supplemented with 10% FBS (Genesee Scientific) and no antibiotics. After 48 h of siRNA KD, each well of cells was washed once with 1× Phosphate-buffered saline (PBS) (Genesee Scientific), trypsinized with 0.1 mL Trypsin (Genesee Scientific) for 2 min at 37 °C, and then resuspended in 0.5 mL of 10% DMEM with no antibiotics for counting and reverse transfection with the same siRNAs and conditions again. siRNA-treated cells were then harvested 24 h post second transfection, for 72 h total of siRNA-mediated KD. siRNA concentrations used for transfections were aimed at reducing target protein or transcript abundance by at least 50% relative to NSC-transfected samples.

Poly I:C (Invivogen, trlr-pic) was forward transfected into Huh7 at 70% confluency, grown in DMEM with 10% FBS, and no antibiotics. Poly I:C was complexed using Lipofectamine 3000 (Thermo Fisher Scientific) without the p3000 reagent in OptiMEM media (Thermo Fisher Scientific), according to the manufacturer’s instructions. The final concentration of Poly I:C transfected was 2 µg/mL and cells were treated for 4 h before harvesting protein for western blot as detailed below.

### Western Blot.

Cells were washed once with 1× PBS before being lysed in ice-cold 1× RIPA buffer (Cell Signaling Technology) supplemented with 1× Phosphatase inhibitor cocktail (Cell Signaling Technology) and 1× Protease inhibitor cocktail (Cell Signaling Technology). Cells were lysed for 30 min on ice, then scraped and supernatant clarified by centrifugation at 10,000×*g* and 4 °C. Total protein was quantified using the BCA assay (Thermo Fisher Scientific), then resuspended in 1× LDS buffer (Genesee Scientific) supplemented with β-mercaproethanol (Millipore-Sigma) before boiling at 95 °C for 10 min. Between 15 to 30 μg of total protein was loaded in each lane. Samples were separated using Bis-tris gradient (4 to 12%) or 10% polyacrylamide gels (Genesee Scientific) in 1× MOPS buffer. Chameleon Duo protein molecular weight marker ladder was loaded alongside samples (Li-cor biosciences). Gels were transferred at 4 °C to a nitrocellulose membrane (Bio-Rad) using the X-Cell blot 2 module (Thermo Fisher Scientific), according to the manufacturer’s protocol in 1× Tris-Glycine transfer buffer supplemented with 20% V/V methanol. Transfer and equal loading were confirmed by Ponceau staining (Cell Signaling Technology). Membranes were blocked for 30 min at RT using Intercept tris-buffered saline (TBS) blocking buffer (Li-cor Biosciences) before overnight incubation with agitation at 4 °C with primary antibodies diluted in TBS blocking buffer. A list of antibodies used, and their dilutions, is in *SI Appendix*, Fig. S13*B*. Following overnight incubation, membranes were washed three times with 1× TBS +0.1% Tween-20. Membranes were incubated for 45 min at RT with IR-Dye secondary antibodies diluted 1:10,000 (Li-cor 926-68072 and 926-32213), then washed three times again with 1× TBS+ 0.1% Tween 20. Membranes were visualized using the Li-Cor Odyssey scanner.

### RNA Isolation and qRT-PCR.

Cells were washed once with 1× PBS before being lysed in RA1 buffer (Macherney Nagel) supplemented with β-mercaproethanol (Millipore-Sigma). RNA was extracted using the NucleoSpin RNA miniprep kit (Macherney Nagel) according to the manufacturer’s protocol. cDNA was synthesized using the high-capacity cDNA reverse transcription kit (Thermo Fisher Scientific) with RNAse inhibitors according to the manufacturer’s protocol. cDNA was diluted 1 in 5 and used for qPCR using the iTaq Universal SYBR Green Supermix (Bio-Rad) in a 10 μL reaction. The reaction mix contained 300 nM of forward and reverse primers. qPCR was performed on the BioRad CFX384 Real Time System instrument using cycling conditions recommended by the iTaq Universal SYBR Green Supermix protocol (Bio-Rad). GAPDH RNA levels were used to normalize C_T_ values using the delta- delta C_T_ method (*SI Appendix*, Fig. S13*C*). qRT-PCR primers to detect GAPDH were from ref. 66 pre-tRNA-Tyr-GTA, *BCRNY1*, *RMRP* were from ref. 67.

### Conditioned Media Experiments for Multiplex Immunoassay and IL6 Receptor Blockage.

Cell culture media were harvested from Huh7 WT and YBX1 KO cells grown to 90% confluency over 3 d. Cell culture media were also harvested from Huh7 WT cells treated with NSC and an siRNA against YBX1 at the 72 h endpoint of siRNA KD. All cell culture media were spun twice at 500×*g* at 4 °C to pellet and remove cells. Then, cell culture media were spun at 14,000×*g* at 4 °C to pellet and remove debris. Clarified media were used for supernatant transfer experiments onto WT Huh7 cells or for analysis using a custom procartaplex immunoassay panel (Thermo Fisher Scientific), analyzed on a MagPix instrument. Procartaplex immunoassays were performed according to the manufacturer’s protocol by the University of Virginia Flow Cytometry Core.

For IL6R signaling blockage experiments, Huh7 WT cells were seeded in DMEM containing 2% FBS and no antibiotics in a 24-well plate format. The next day, an IL6R blocking antibody- Tocilizumab (MedChemExpress, HY-P9917) was diluted in 1× PBS to a 6× concentrated stock before 100 μL was added to appropriate wells and incubated at 37 °C, 5% CO_2_ for 1 h, gently rocking every 15 mins. Following, 500 μL of clarified conditioned media from Huh7 WT cells treated with an NSC siRNA or an siRNA against YBX1 was added on top of wells containing 6× concentrated Tocilizumab or PBS alone. Alternatively, 500 μL DMEM containing 10% FBS, no antibiotics, and 5 ng/mL of recombinant human interleukin 6 protein (rhIL6, R&D Systems, 206-IL-010) was also added to wells to test Tocilizumab efficacy. The final concentration of Tocilizumab in each well was 10 μg/mL. Cells were then incubated for an additional 4 h at 37 °C, 5% CO_2_ before harvesting proteins for western blot as detailed above.

### RNA Sequencing.

RNA was extracted using Trizol (Thermo Fisher Scientific) according to the manufacturer’s protocol. Ribosomal RNA was removed from the RNA sample using the NEBNext ribosomal RNA depletion kit (NEB) and then stranded RNA-Seq library prepared using the NEBNext ultra II stranded RNA library preparation kit (NEB) following the manufacturer’s recommended procedure. The pooled library was sequenced on Illumina NextSeq 550 for paired end 75 bp reads. The reads are aligned to human reference genome GRCh38 and quantified for gene level expression using STAR version 2.7.10a. The counts were normalized and differential expression detected using DESeq2.

### GSEA.

GSEA was performed on Log2 normalized counts of RNA Seq data of YBX1 KO and WT cells. Genes were ranked using the differences of the classes method. GSEA was performed using the MSigDB Hallmark gene sets.

For cross-species GSEA of data from Jayavelu et al. (28), we converted all DEGs identified during murine YBX1 KD (with their associated Log2 fold changes) to human orthologs using ENSEMBL. Then, this list was used for analysis with preranked GSEA, using the same human hallmark MSigDB gene sets to compare to our data. DEGs identified during YBX3 KD from Cooke et al. (29) (with their associated Log2 fold changes) were used for analysis with preranked GSEA with MSigDB hallmark gene sets to compare to our data. Volcano plots of DEGs were generated using the EnhancedVolcano R package (68). GSEA pathway plots were generated using ggPlot2 (69).

### Statistical Analysis.

For all data generated, normality was assessed using Q–Q plots. Comparisons between two groups were performed using unpaired Student’s *t* tests. Comparisons among more than two groups were performed using one-way ANOVA with Tukey’s post hoc test. For experiments involving two independent variables, statistical analyses were performed using two-way repeated-measures ANOVA followed by Šídák’s multiple comparisons test. Statistical analyses were performed using Prism V10 software (GraphPad).

## Supplementary Material

Appendix 01 (PDF)

Dataset S01 (XLSX)

Dataset S02 (XLSX)

Dataset S03 (XLSX)

## Data Availability

Fastq files from YBX1 RNA-Seq experiments are deposited in the Gene Expression Omnibus database under GSE310735 (70). All other data are included in the manuscript and/or supporting information.
