# Supplementary material for: YBX1 regulates RNA polymerase III transcripts to prevent inflammation
Source: Proc Natl Acad Sci U S A. 2026 Aug 21;123(35):e2605741123. doi: 10.1073/pnas.2605741123 (PMC13535238; doi:10.1073/pnas.2605741123)
Supplement: Supplementary file 1 [file pnas.2605741123.sapp.pdf]

Supporting Information for
YBX1 regulates RNA polymerase III transcripts to prevent inflammation

Tania I. Strilets[1,2]*, Sarah E. Dremel[2,3], Mariano A. Garcia-Blanco[2,3]*

Mariano A. Garcia-Blanco
Email: marianogb@virginia.edu

This PDF file includes:

Supplementary Figures 1- 13

Other supporting materials for this manuscript include the following:

Datasets S1 to S3

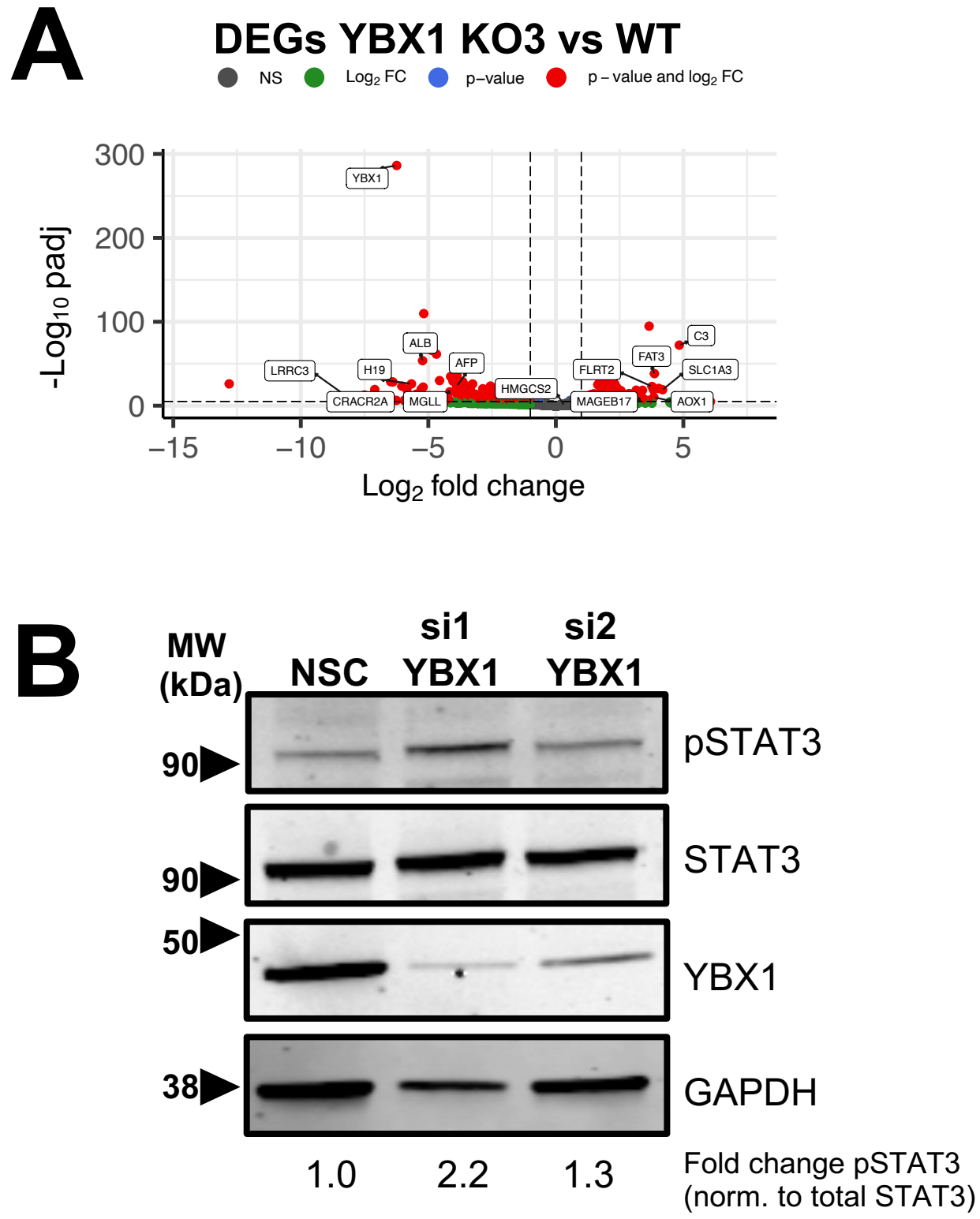

Supplementary Figure 1. Further characterization of phenotypes in YBX1 KO and KD cells
(A) Volcano plot of differentially expressed genes (DEGs) in Huh7 YBX1 knock-out clone 3 (KO3) versus wild-type (WT) cells, analyzed using DeSeq2. The top seven upregulated and downregulated genes common to two YBX1 KO clones are annotated. Log2 fold change cutoff=1, adjusted p-value cutoff= $\leq 1\times10^{-5}$. Between the two YBX1 KO clones, there are 58 common DEGs downregulated $\leq$ -2-fold relative to WT cells, and 92 common DEGs upregulated by more than $\geq$ 2-fold (padj cutoff $\leq$ 0.05).
(B) Western blot of phosphorylated STAT3 (pSTAT3), total STAT3, YBX1, and GAPDH protein levels in Huh7 WT treated with non-silencing control (NSC) siRNA or two siRNAs against YBX1 (si1, si2 YBX1). Band intensity of pSTAT3 was determined by densitometric analysis in ImageJ, normalized to total STAT3, and expressed as fold change relative to NSC cells. Due to GAPDH loading differences, the fold change of pSTAT3 was also quantified relative to GAPDH, whose levels were 4.3- and 1.3-fold higher in NSC samples for siRNA1 and siRNA2, respectively.
Data are from one representative experiment.

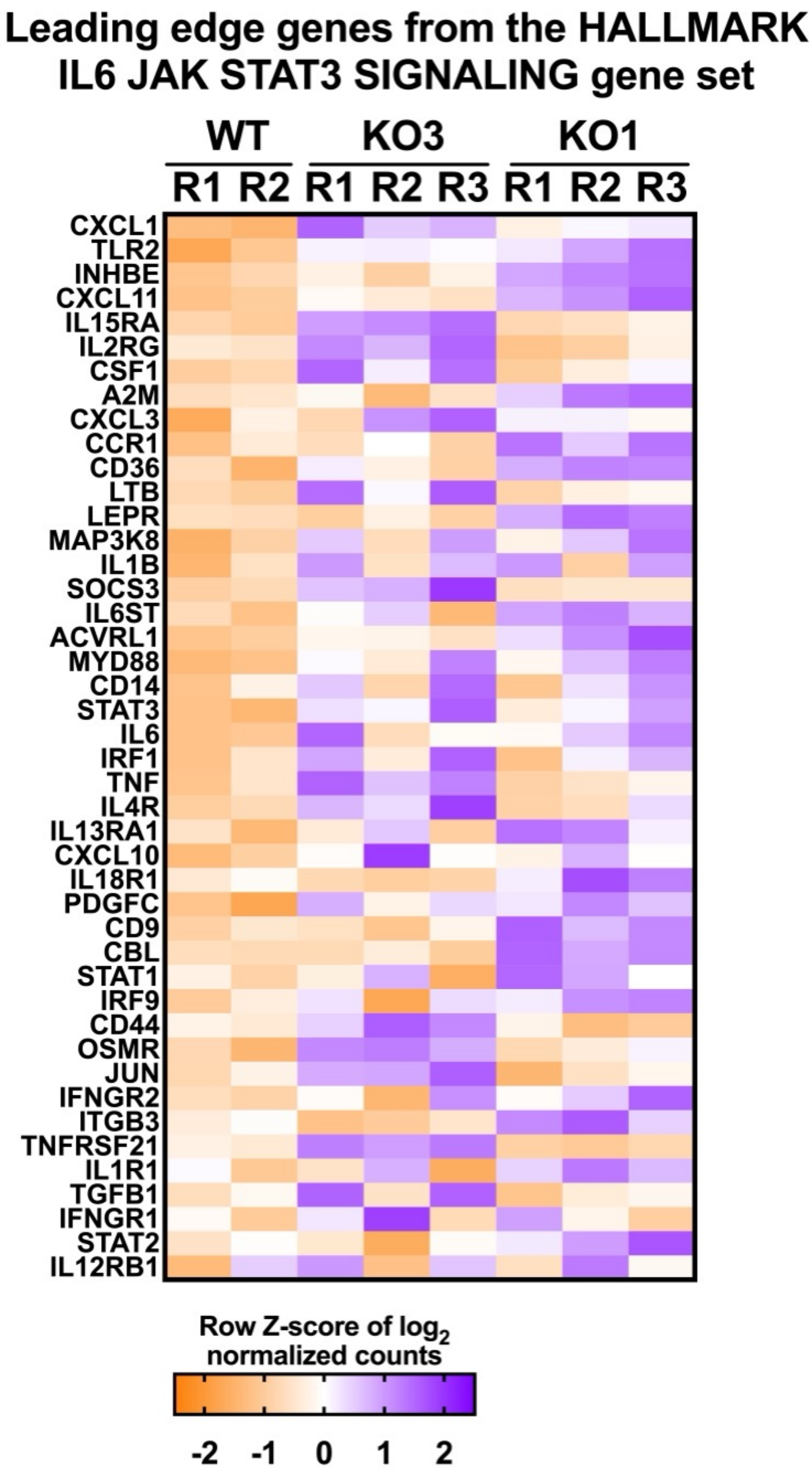

Supplementary Figure 2. Leading-edge genes contributing to the positive enrichment of the hallmark "IL6 JAK STAT3 SIGNALING" gene set in YBX1 KO cells
Heatmaps showing row z-scores of log2-normalized RNA-Seq counts in Huh7 wild-type (WT) and YBX1 KO cells (KO3, KO1). Heatmaps were created using GraphPad Prism software.

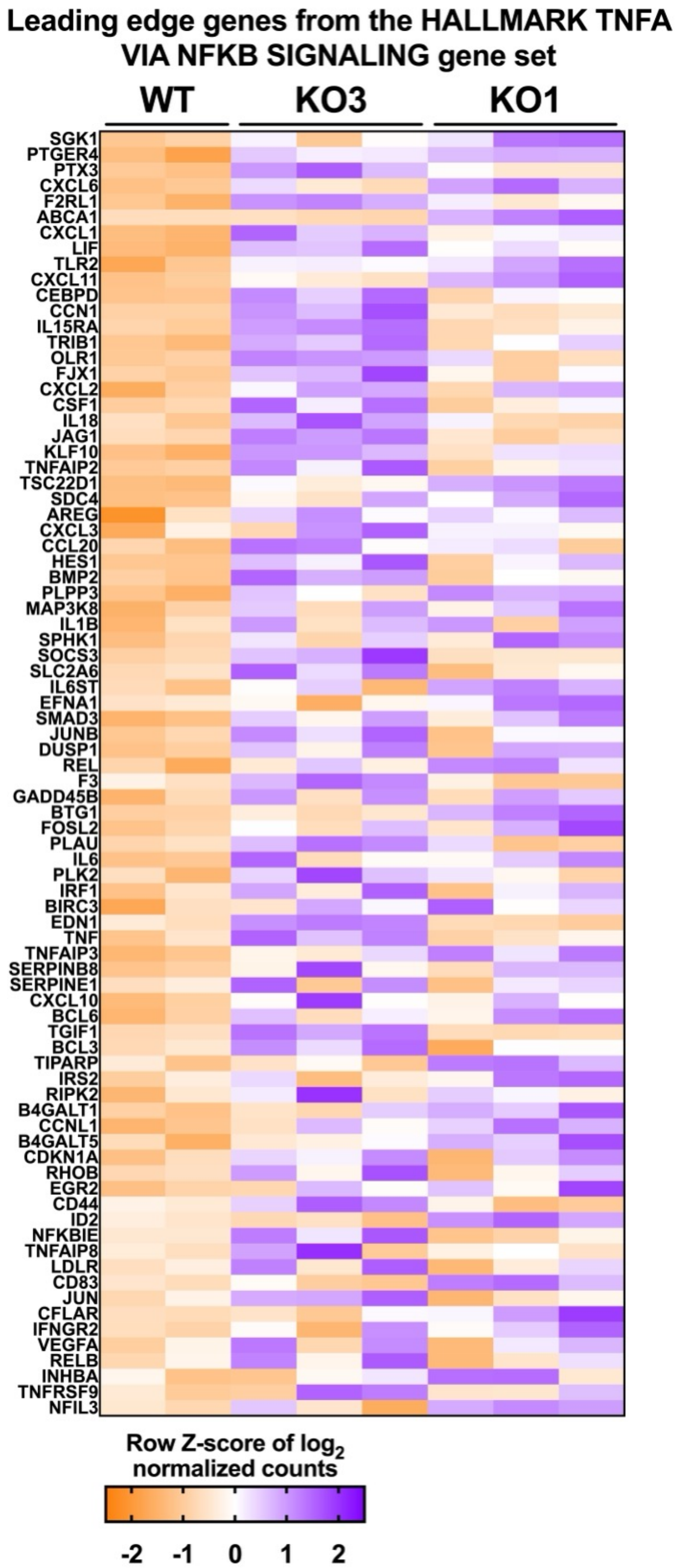

Supplementary Figure 3. Leading-edge genes contributing to the positive enrichment of the hallmark "TNFA SIGNALING VIA NFKB" gene set in YBX1 KO cells

Heatmaps showing row z-scores of log2-normalized RNA-Seq counts in Huh7 wild-type (WT) and YBX1 KO cells (KO3, KO1). Heatmaps were created using GraphPad Prism software.

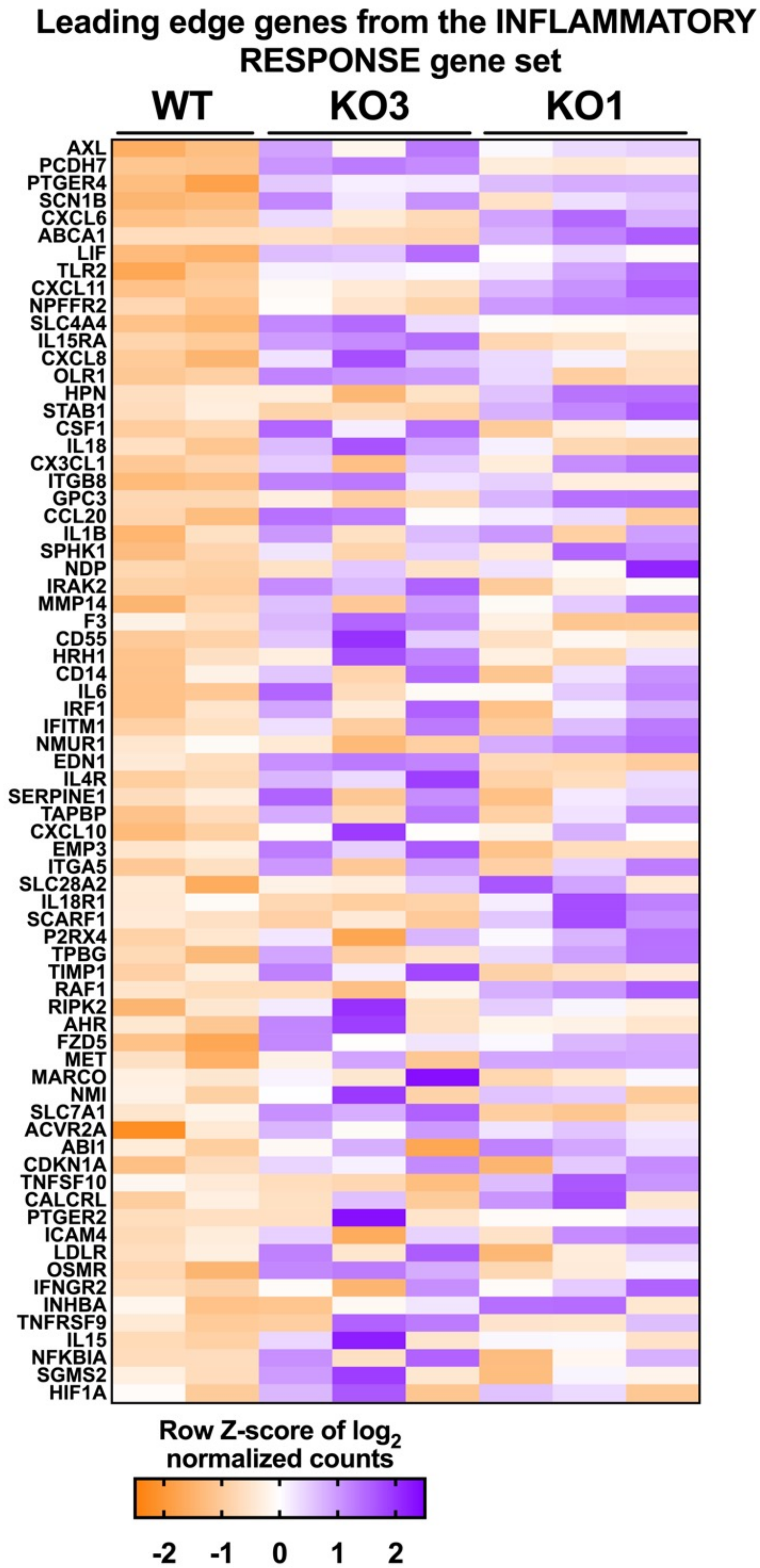

Supplementary Figure 4. Leading-edge genes contributing to the positive enrichment of the hallmark “INFLAMMATORY RESPONSE” gene set in YBX1 KO cells
Heatmaps showing row z-scores of log2-normalized RNA-Seq counts in Huh7 wild-type (WT) and YBX1 KO cells (KO3, KO1). Heatmaps were created using GraphPad Prism software.

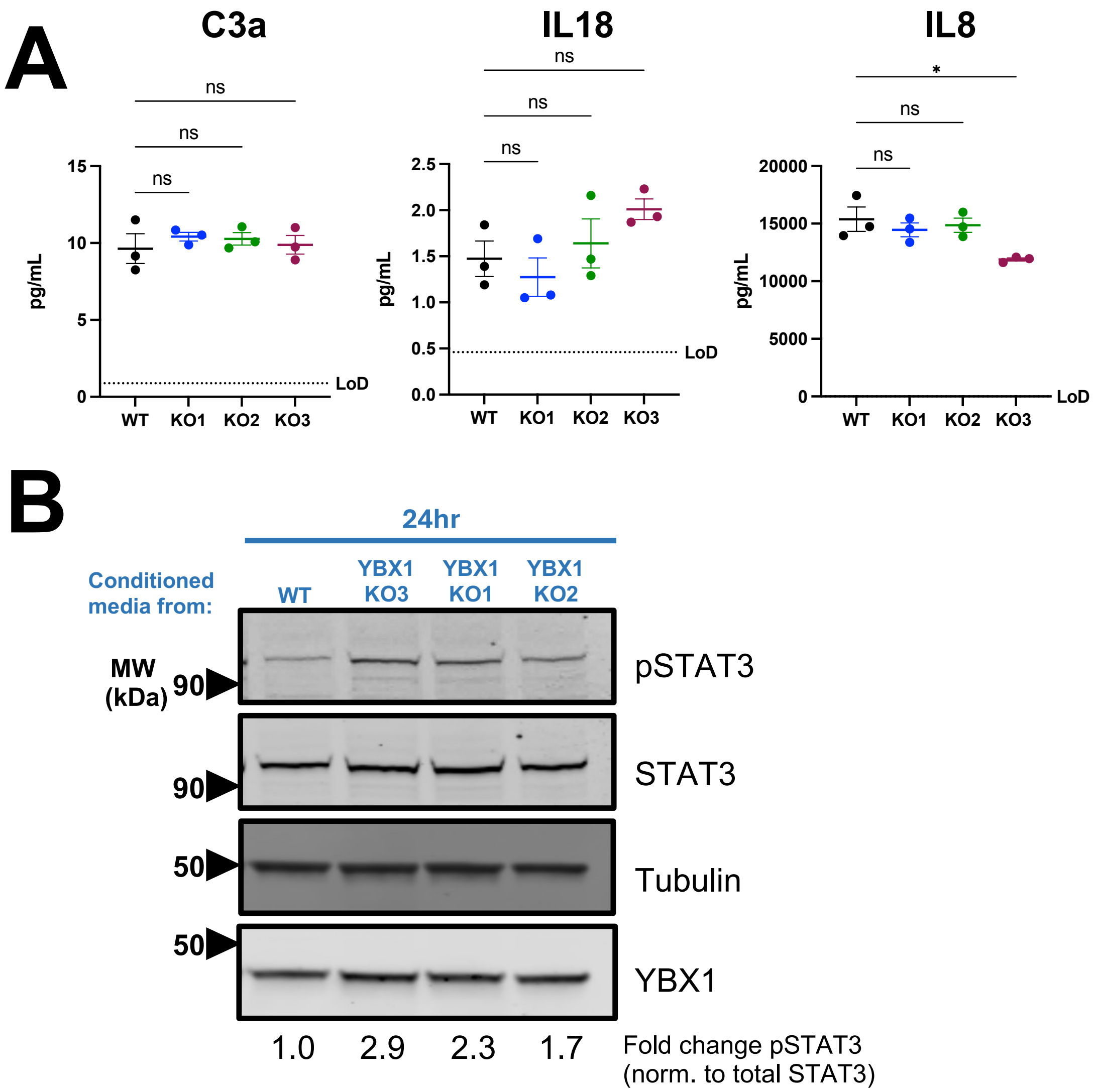

Supplementary Figure 5. Conditioned media from YBX1 KO and KD cells do not secrete all pro-inflammatory cytokines identified as upregulated by transcriptomic analysis
(A) Multiplexed immunoassay results of conditioned media harvested from Huh7 WT and YBX1 KO cells to detect secreted cytokines and pro-inflammatory mediators. * =p<0.05, ** = p<0.005, ***=p<0.0005, ****=p<0.00005, ns= not significant. Lines represent mean ± S.E.M. LoD = Limit of detection pg/mL = picograms per millilitre. Data represent triplicate values from one independent experiment.
(B) Conditioned media from Huh7 WT and YBX1 KO cells were used to culture Huh7 WT cells for 24hr before harvesting cells for western blot to assay STAT3 phosphorylation. Band intensity of pSTAT3 was determined by densitometric analysis in ImageJ, normalized to total STAT3, and displayed as fold change relative to WT cells. Data are from one representative experiment.

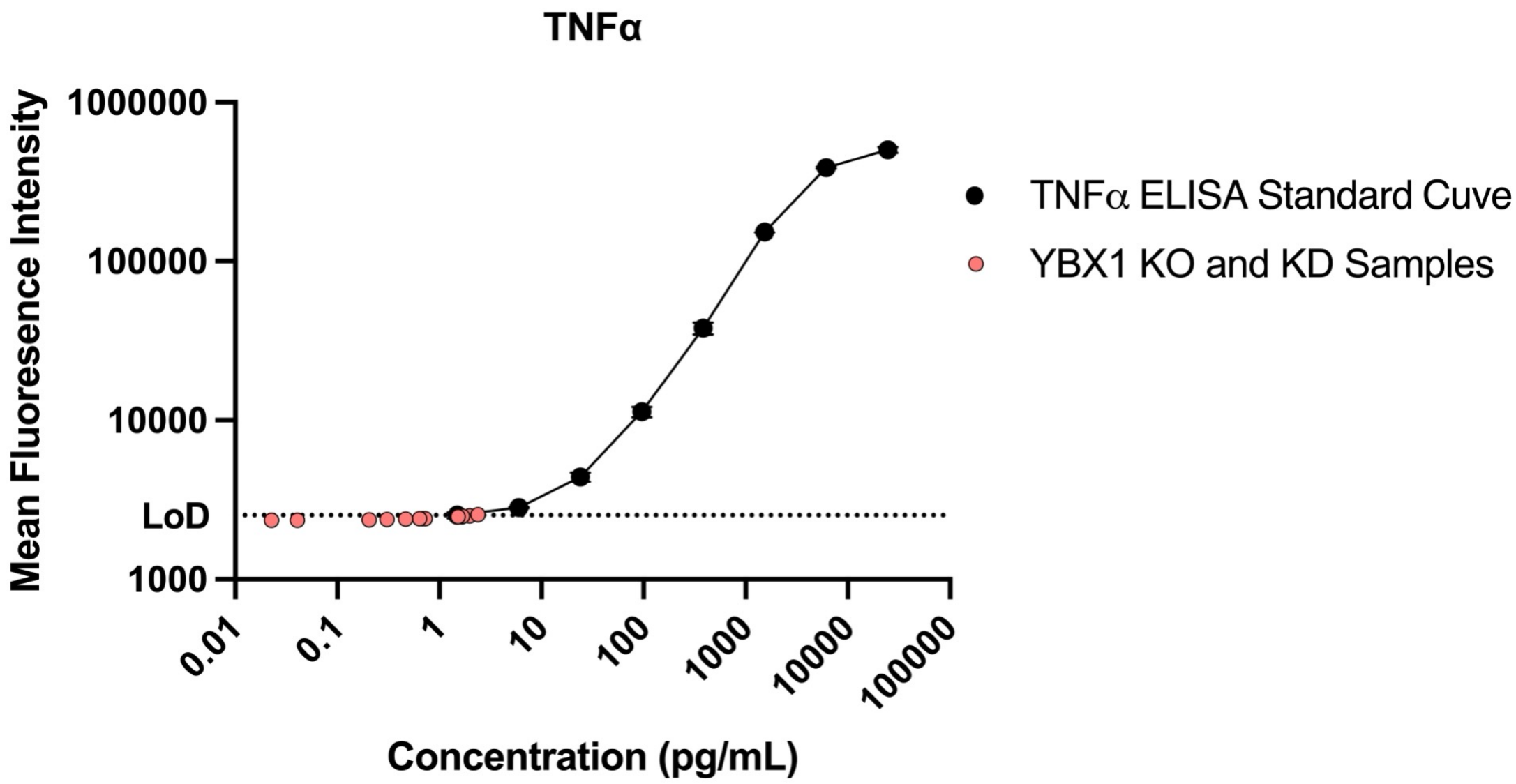

Supplementary Figure 6. No detection of TNFα secretion in YBX1 KO and KD cell supernatants

Multiplexed immunoassay results of conditioned media harvested from Huh7 WT and YBX1 KO cells to detect secreted TNF**α**. TNFα standard curve shown for reference. Each dot represents the mean fluorescence intensity of technical duplicates. LoD = Limit of detection pg/mL = picograms per millilitre. Data are from one independent experiment.

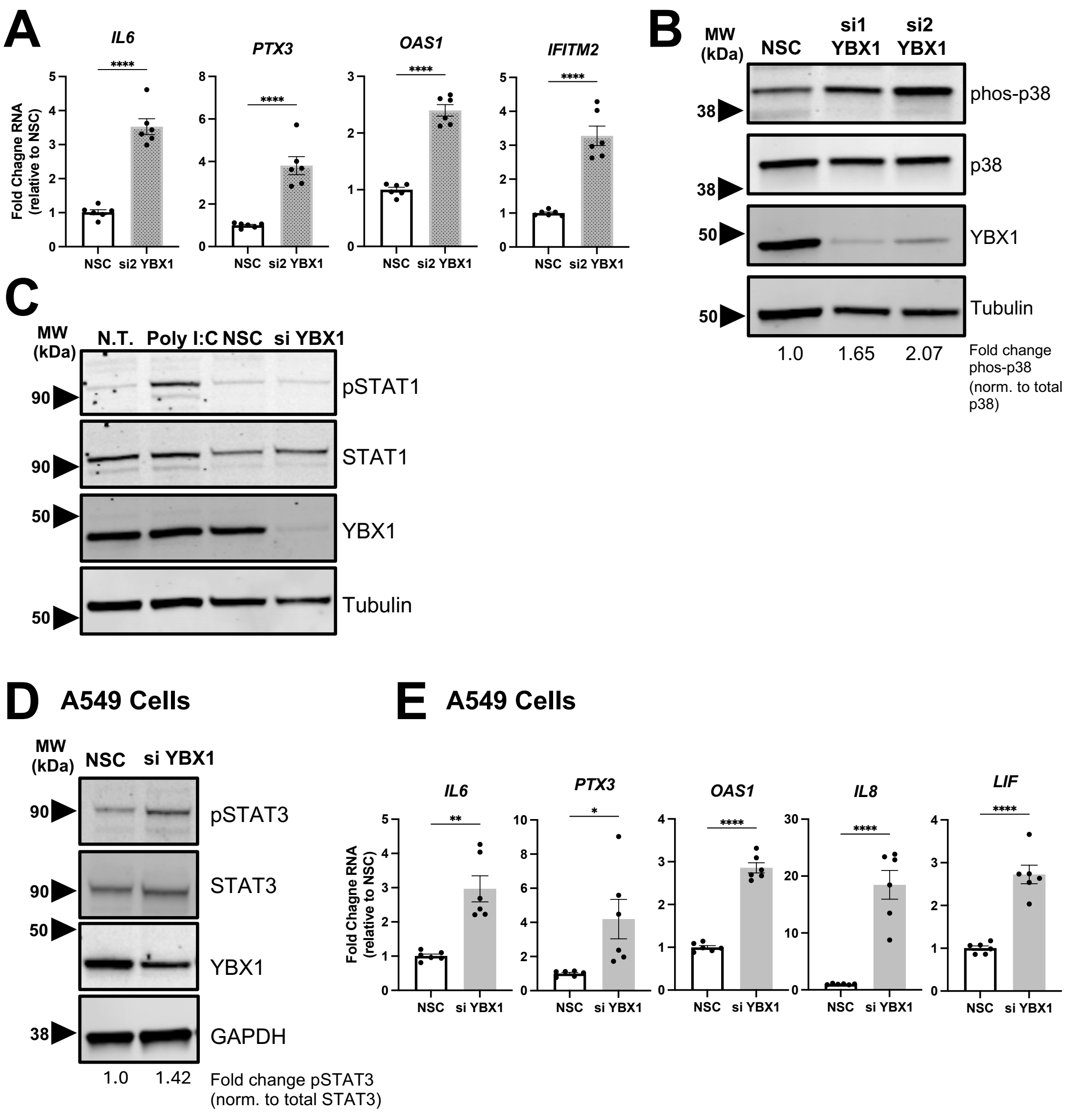

Supplementary Figure 7. IL6/STAT3 and ISG pro-inflammatory phenotype associated with YBX1 depletion is not specific to Huh7 cells

(A) qRT-PCR of select pro-inflammatory transcripts identified as upregulated in YBX1 KO cells via RNA-Seq during YBX1 knockdown (KD) in Huh7 WT cells treated with non-silencing control (NSC) siRNA, a second siRNA against YBX1 (si2 YBX1). For all qRT-PCR data in this figure, transcripts were normalized to *GAPDH* and expressed as fold change relative to NSC-treated cells. Bars represent mean $\pm$ S.E.M. * =p<0.05, ** = p<0.005, ***=p<0.0005, ****=p<0.00005, ns= not significant. Each point represents one experimental sample. Data represent triplicate values from two independent experiments.

(B) Western blot of phosphorylated p38 (phos-p38), total p38, YBX1, and tubulin protein levels in Huh7 WT cells treated with non-silencing control (NSC) siRNA or two siRNAs against YBX1 (si1, si2 YBX1). Band intensity of phos-p38 was determined by densitometric analysis in ImageJ, normalized to total p38, and expressed as fold change relative to NSC-treated cells. Data are from one representative experiment.

(C) Western blot of phosphorylated STAT1 (pSTAT1), total STAT1, YBX1, and tubulin Huh7 WT cells untreated (N.T.), treated with 2 µg/mL of Poly I: C for 4 hours, or with non-silencing control

(NSC) siRNA or an siRNA against YBX1 (si1 YBX1). Band intensities for pSTAT1 are shown as fold changes relative to N.T cells or NSC cells. Data are from one representative experiment.

(D) Western blot of phosphorylated STAT3 (pSTAT3), total STAT3, YBX1, and GAPDH protein levels in A549 cells treated with a non-silencing control (NSC) siRNA or an siRNA against YBX1 (si YBX1). Data are from one representative experiment. Band intensity of pSTAT3 was determined by densitometric analysis in ImageJ, normalized to total STAT3, and expressed as fold change relative to NSC cells.

(E) qRT-PCR of select pro-inflammatory transcripts upregulated in A549 cells treated with a non-silencing control (NSC) siRNA or an siRNA against YBX1 (si YBX1).

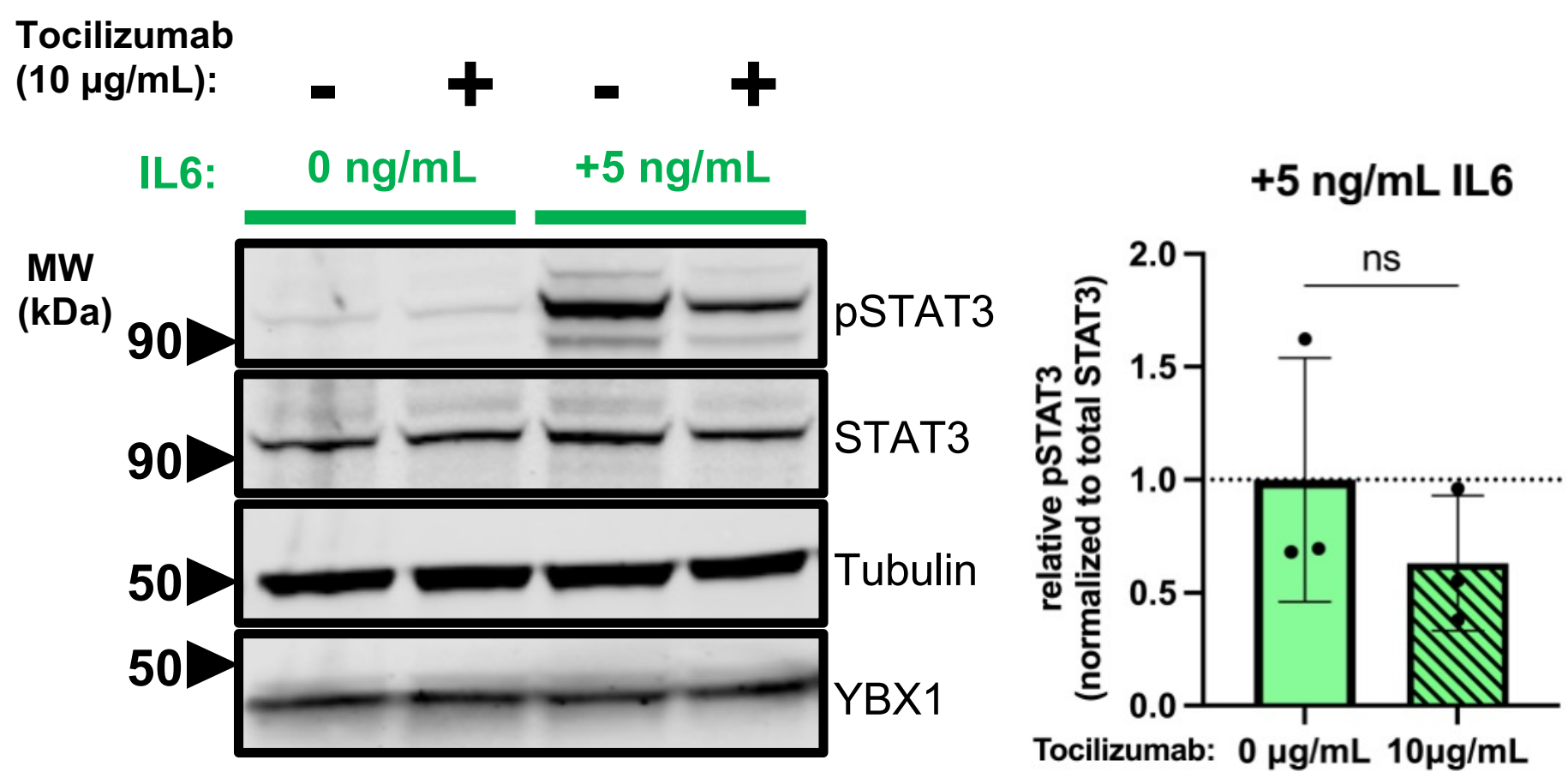

Supplementary Figure 8. Testing Tocilizumab Efficacy
Huh7 cells were pretreated with an anti-IL6 receptor antibody (Tocilizumab, 10 µg/mL) for 1 hour at 37°C before the addition of recombinant human IL6 (5 ng/mL) for 4 hours, and then processed for western blotting of pSTAT3, total STAT3, Tubulin, and YBX1. Western blot data are from one representative experiment. pSTAT3 band intensity was determined by densitometric analysis in ImageJ, normalized to total STAT3, and expressed as fold change relative to cells treated with no Tocilizumab. ns= not significant as determined by a Mann-Whitney test. Quantified pSTAT3 data are from three independent experiments.

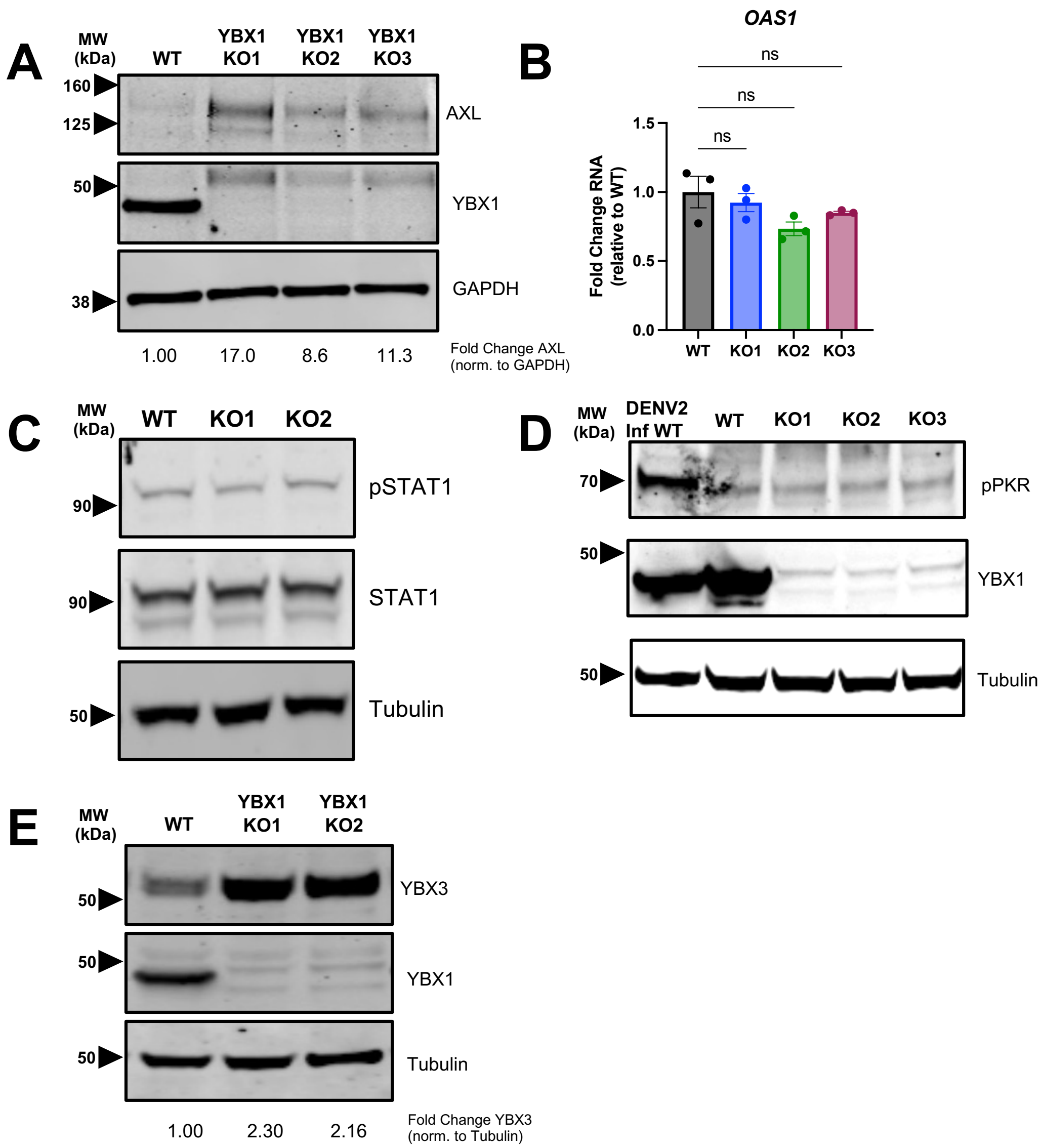

Supplementary Figure 9. Further characterization of YBX1 KO and KD cells

(A) Western blot of AXL protein expression in Huh7 WT and YBX1 KO clonal cell lines (KO1, KO2, KO3). AXL band intensity was determined by densitometric analysis in ImageJ, normalized to GAPDH, and expressed as fold change relative to WT cells. All western blot data in this figure are from one representative experiment.

(B) qRT-PCR of *OAS1* in YBX1 KO cells. Transcripts were normalized to *GAPDH* and expressed as fold change relative to WT cells. ns= not significant. Data represent triplicate values from one independent experiment. Bars represent mean $\pm$ S.E.M.

(C) Western blot of phosphorylated STAT1 (pSTAT1), total STAT1, YBX1, and tubulin protein levels in Huh7 WT and YBX1 KOs.

(D) Western blot of phosphorylated PKR (pPKR), YBX1, and tubulin protein levels in Huh7 WT and YBX1 KOs. Huh7 WT cells infected with Dengue virus 2 (New Guinea C strain) at a multiplicity of infection of 10 for 72 hours were used as a positive control for PKR phosphorylation via western blot (DENV-2 Inf WT).

(E) Western blot of YBX3 protein expression in Huh7 WT and YBX1 KOs. Band intensity of YBX3 was determined by densitometric analysis in ImageJ, normalized to Tubulin, and expressed as fold change relative to WT cells.

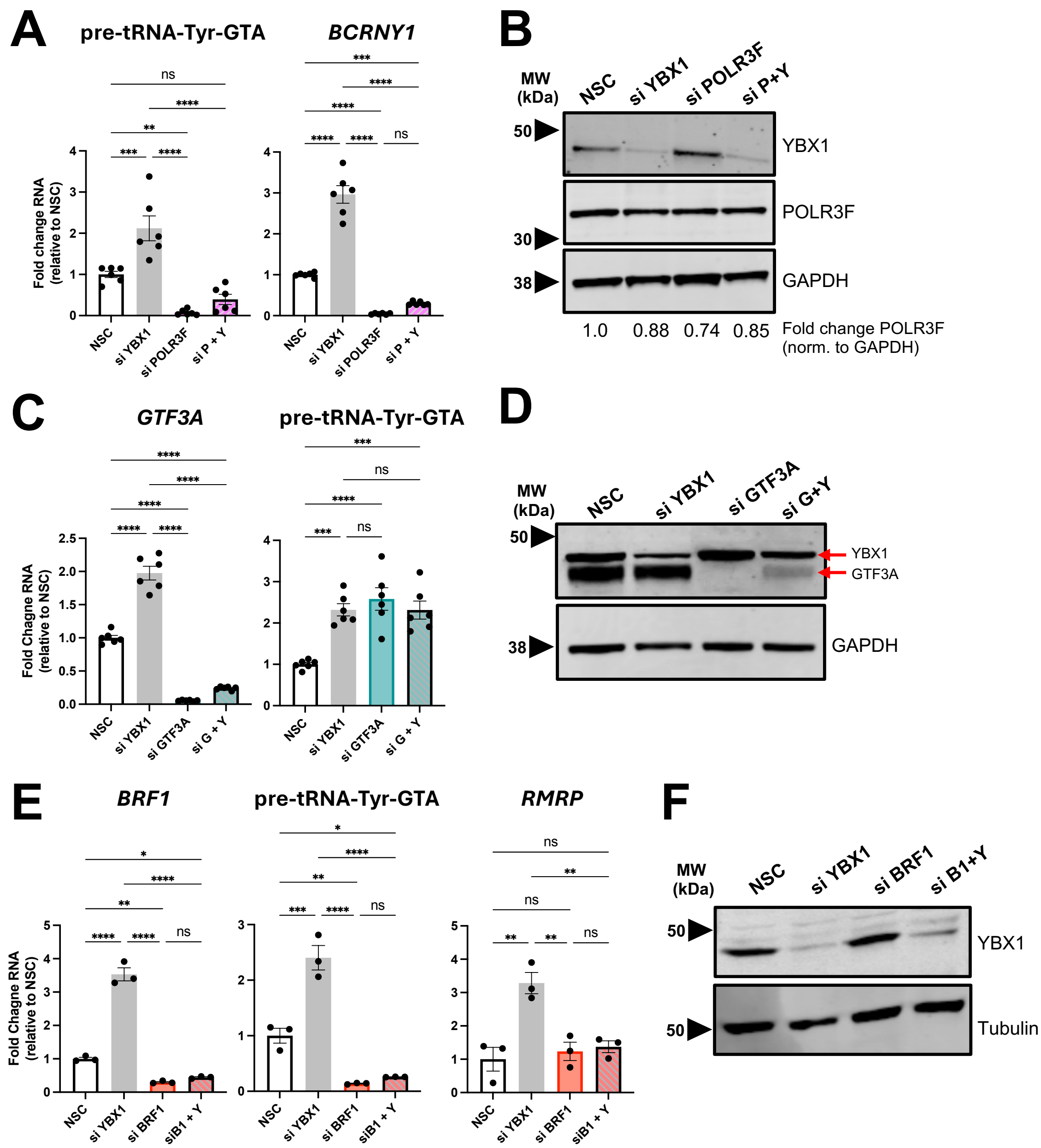

Supplementary Figure 10. Validation of RNAP III transcription factor knockdown

(A) qRT-PCR assaying levels of RNAP III transcripts (pre-tRNA-Tyr-GTA and *BCRNY1*) during during POL3RF and YBX1 knock down. Huh7 WT cells were treated with non-silencing control (NSC) siRNA, an siRNA against YBX1 (si YBX1), an siRNA pool against POLR3F (si POLR3F), or a combination of an siRNA against YBX1 and an siRNA pool against POLR3F (siP + Y). Data are from two independent experiments. For all qRT-PCR data in this figure, transcripts were normalized to *GAPDH* and expressed as fold change relative to NSC-treated cells. Each point represents one experimental sample. * =p<0.05, ** = p<0.005, ***=p<0.0005, ns= not significant. Bars represent mean ± S.E.M.

(B) Western blot to assess siRNA-mediated knockdown of both YBX1 and POLR3F during co-knockdown experiments. Band intensity of POLR3F was determined by densitometric analysis in ImageJ, normalized to GAPDH protein levels, and expressed as fold change relative to NSC cells. Data are from one representative experiment.

(C) qRT-PCR assaying levels of *GTF3A* alongside type 2 RNAP III transcripts (pre-tRNA-Tyr-GTA) during GTF3A and YBX1 knock down. Huh7 WT cells were treated with non-silencing control (NSC) siRNA, an siRNA against YBX1 (si YBX1), an siRNA pool against GTF3A (si GTF3A), or a combination of an siRNA against YBX1 and an siRNA pool against GTF3A (siG + Y). Data are from two independent experiments.
(D) Western blot to assess siRNA-mediated knockdown of both YBX1 and GTF3A during co-knockdown experiments. Data are from one representative experiment.
(E) qRT-PCR assaying levels of *BRF1*, alongside type 2 and 3 RNAP III transcripts (pre-tRNA-Tyr-GTA and *RMRP*, respectively) during BRF1 and YBX1 knock down. Huh7 WT cells were treated with non-silencing control (NSC) siRNA, an siRNA against YBX1 (si YBX1), an siRNA pool against BRF1 (si BRF1), or a combination of an siRNA against YBX1 and an siRNA pool against BRF1 (siB1 + Y). Data represent triplicate values from one independent experiment.
(F) Western blot to assess siRNA-mediated knockdown of YBX1 during co-knockdown experiments with BRF1. Data are from one representative experiment.

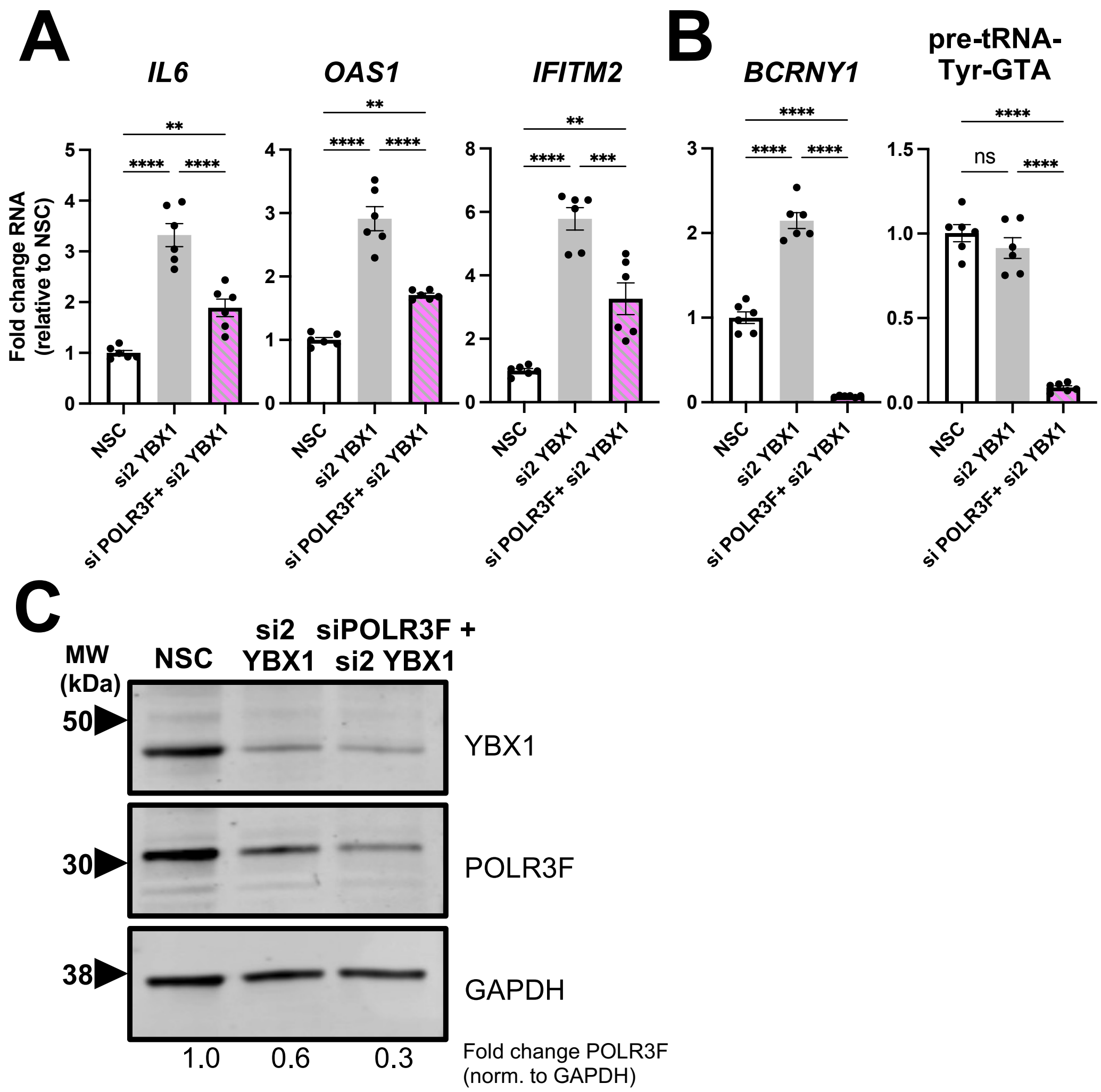

Supplementary Figure 11. Co-depletion of YBX1 and POLR3F with a second siRNA against YBX1

(A) qRT-PCR assaying the upregulation of *IL6, OAS-1, and IFITM2* during simultaneous knockdown of YBX1 and RNAP III machinery. Huh7 WT cells were treated with non-silencing control (NSC) siRNA, a second siRNA against YBX1 (si2 YBX1), or a combination of the second siRNA against YBX1 and an siRNA against POLR3F (si POLR3F + si2 YBX1). For all qRT-PCR data in this figure, transcripts were normalized to *GAPDH* and expressed as fold change relative to NSC-treated cells. * =p<0.05, ** = p<0.005, ***=p<0.0005, ****=p<0.00005, ns= not significant. Each point represents one experimental sample. Data are from two independent experiments. Bars represent mean ± S.E.M.

(B) Same as in (A), but assaying levels of RNAP III transcripts (pre-tRNA-Tyr-GTA and *BCRNY1*) during YBX1 and POLR3F knockdown.

(C) Western blot to assess siRNA-mediated knockdown of both YBX1 and POLR3F during co-knockdown experiments in (A, B). Band intensity of POLR3F was determined by densitometric analysis in ImageJ, normalized to GAPDH protein levels, and expressed as fold change relative to NSC cells. Data are from one representative experiment.

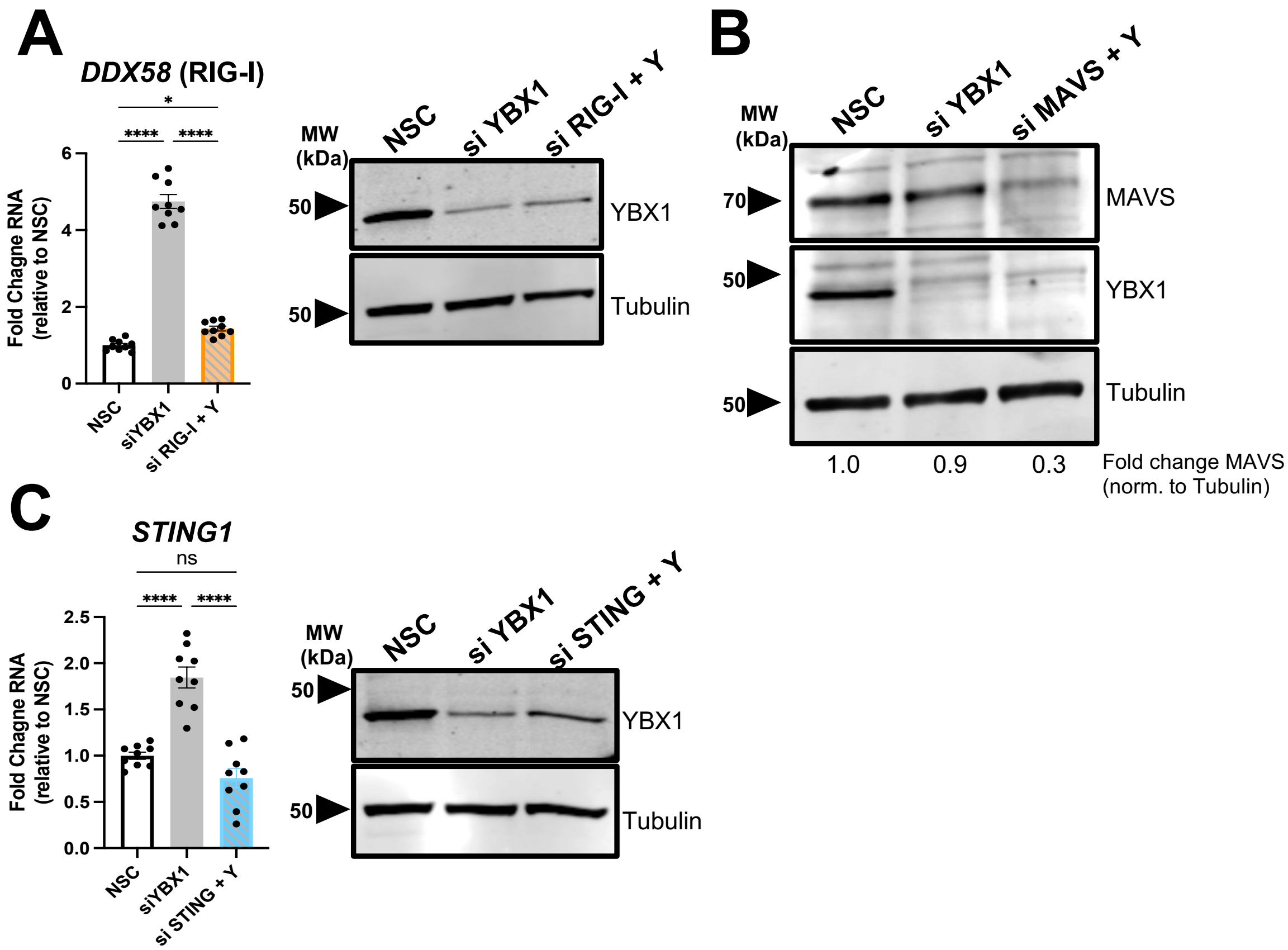

Supplementary Figure 12. Validation of RIG-I, MAVS, and STING knockdown

(A) qRT-PCR assaying *DDX58* (RIG-I) levels during RIG-I and YBX1 knockdown. Huh7 WT cells were treated with non-silencing control (NSC) siRNA, an siRNA against YBX1 (si YBX1), or a combination of an siRNA against YBX1 and an siRNA pool against RIG-I (si RIG-I + Y). Transcripts were normalized to *GAPDH* and expressed as fold change relative to NSC-treated cells. * =p<0.05, ns= not significant. Each point represents one experimental sample. qRT-PCR data are from three independent experiments. Bars represent mean $\pm$ S.E.M. Western blot was used to assess siRNA-mediated knockdown of YBX1 during co-knockdown with RIG-I. Western blot data are from one representative experiment.

(B) Western blot to assess siRNA-mediated knockdown of both YBX1 and MAVS during co-knockdown experiments. Data are from one representative experiment.

(C) Same as in **(A)** but for the co-knockdown of YBX1 and STING.

A

siRNA Target	Catalog Number	Final concentration
Non-silencing control (NSC)	4390843 (Thermo Fisher Scientific)	30 - 130nM
YBX1 si1	Hs_YBX1_1 Flexitube siRNA (Qiagen)	30 nM
YBX1 si2	Hs_YBX1_7 Flexitube siRNA (Qiagen)	30 nM
POLR3F siPool	L-019240-01-0005 (Dharmacon)	30 nM
GTF3A siPool	L-012635-00-0005 (Dharmacon)	30 nM
BRF1 siPool	L-017422-00-0005 (Dharmacon)	80 nM
RIG-I siPool	L-012511-00-0005 (Dharmacon)	100nM
MAVS siPool	L-024237-00-0005 (Dharmacon)	100nM
STING siPool	L-024333-00-0005 (Dharamacon)	100nM

B

Antibody Target	Catalog Number	Dilution used
GAPDH	GTX627408 (GeneTex)	1: 5000
Tubulin	3873S (Cell Signaling Technology)	1: 2000
YBX1	702245 (Thermo Fisher Scientific)	1: 2000
pSTAT3	9145S (Cell Signaling Technology)	1: 500
STAT3	4904T (Cell Signaling Technology)	1: 2000
pNF-kB	MA5-15160 (Thermo Fisher Scientific)	1:500
NF-kB	8242S (Cell Signaling Technology)	1:1000
phospho p38	9211S (Cell Signaling Technology)	1: 500
p38	9212S (Cell Signaling Technology)	1: 1000
AXL	8661S ((Cell Signaling Technology)	1: 500
YBX3	A303-070A (Thermo Fisher Scientific)	1: 500
pSTAT1	9167S (Cell Signaling Technology)	1: 500
STAT1	9172T (Cell Signaling Technology)	1: 1000
pPKR	ab32036 (Abcam)	1: 500
POLR3F	ab180501 (Abcam)	1: 500
GTF3A	ab129440 (Abcam)	1: 500
MAVS	PA5-17256 (Thermo Fisher Scientific)	1: 1000

C

Target	Sequence (5' to 3')
IL6 Forward	GGAGACTTGCCTGGTGAAA
IL6 Reverse	CTGGCTTGTTCCTCACTACTC
LIF Forward	TGTCGCTCTCTAAGCACTTTA
LIF Reverse	GGATGGACAGATGGACAACTC
PTX3 Forward	GTGGTGGCTTTGATGAAACATT
PTX3 Reverse	CCTCCGGTCTCTCTTATCTCTT
OAS1 Forward	ATAAAAGCAAACAGGTCTGG
OAS1 Reverse	CTGGCAAGAGATAGTCTTC
IFITM2 Forward	CGATCACCGCTGGTCACCATGAA
IFITM2 Reverse	CCCGGTCACGTCGCCAACC
IL8 Forward	GGACAAGAGCCAGGAAGAAA
IL8 Reverse	ACACAGAGCTGCAGAAATCA
GTF3A Forward	TGACTGCAGCGCCAATTA
GTF3A Reverse	CTTGCCACACCCTTCATAGT
BRF1 Forward	CGGCATCTACAAGGAACACA
BRF1 Reverse	CTTCTTCTGCTCCAGCATCTT

Supplementary Figure 13. Reagents used in this study
(A) Targets, concentrations, and catalog numbers of siRNAs used in this study.
(B) Targets, dilutions, and catalog numbers of Antibodies used in this study.
(C) Sequences of qRT-PCR primers used in this study.

Dataset S1 (separate file). Input data and Gene Set Enrichment Analysis results for YBX1 KO cells.

Dataset S2 (separate file). Input data and Gene Set Enrichment Analysis results for meta-analysis of (1).

Dataset S3 (separate file). Input data and Gene Set Enrichment Analysis results for meta-analysis of (2).

SI References

1. A. K. Jayavelu, *et al.*, Splicing factor YBX1 mediates persistence of JAK2-mutated neoplasms. *Nature* **588**, 157–163 (2020).
2. A. Cooke, *et al.*, The RNA-Binding Protein YBX3 Controls Amino Acid Levels by Regulating SLC mRNA Abundance. *Cell Rep* **27**, 3097-3106.e5 (2019).
